# Stress reactivity as a putative mechanism linking childhood trauma with clinical outcomes in individuals at ultra-high-risk for psychosis: Findings from the EU-GEI High Risk Study

**DOI:** 10.1017/S2045796021000251

**Published:** 2021-05-28

**Authors:** I. Paetzold, I. Myin-Germeys, A. Schick, B. Nelson, E. Velthorst, F. Schirmbeck, J. van Os, C. Morgan, J. Hartmann, M. van der Gaag, L. de Haan, L. Valmaggia, P. McGuire, M. Kempton, U. Reininghaus, Philip McGuire, Lucia R. Valmaggia, Matthew J. Kempton, Maria Calem, Stefania Tognin, Gemma Modinos, Lieuwe de Haan, Mark van der Gaag, Eva Velthorst, Tamar C. Kraan, Nadine Burger, Daniella S. van Dam, Neus Barrantes-Vidal, Tecelli Domínguez-Martínez, Paula Cristóbal-Narváez, Thomas R. Kwapil, Manel Monsonet-Bardají, Lídia Hinojosa, Anita Riecher-Rössler, Stefan Borgwardt, Charlotte Rapp, Sarah Ittig, Erich Studerus, Renata Smieskova, Rodrigo Bressan, Ary Gadelha, Elisa Brietzke, Graccielle Asevedo, Elson Asevedo, Andre Zugman, Stephan Ruhrmann, Dominika Gebhard, Julia Arnhold, Joachim Klosterkötter, Dorte Nordholm, Lasse Randers, Kristine Krakauer, Tanya Louise Naumann, Louise Birkedal Glenthøj, Merete Nordentoft, Marc De Hert, Ruud van Winkel, Barnaby Nelson, Patrick McGorry, Paul Amminger, Christos Pantelis, Athena Politis, Joanne Goodall, Gabriele Sachs, Iris Lasser, Bernadette Winklbaur, Mathilde Kazes, Claire Daban, Julie Bourgin, Olivier Gay, Célia Mam-Lam-Fook, Marie-Odile Krebs, Bart P. Rutten, Jim van Os

**Affiliations:** 1 Department of Public Mental Health, Central Institute of Mental Health, Medical Faculty Mannheim, Heidelberg University, Mannheim, Baden-Württemberg, Germany; 2 Department of Neurosciences, Center for Contextual Psychiatry, KU Leuven, Leuven, Flanders, Belgium; 3 Centre for Youth Mental Health, University of Melbourne, Parkville, Victoria, Australia; 4 Orygen, The National Centre of Excellence in Youth Mental Health, Melbourne, Victoria, Australia; 5 Department of Psychiatry, Icahn School of Medicine at Mount Sinai, New York, NY, USA; 6 Department of Psychiatry, Amsterdam UMC, Location AMC, University of Amsterdam, Amsterdam, North Holland, Netherlands; 7 Arkin, Institute for Mental Health, Amsterdam, North Holland, Netherlands; 8 Department of Psychiatry and Neuropsychology, Maastricht University School for Mental Health and Neuroscience, Faculty of Health, Medicine and Life Sciences, Maastricht, Limburg, Netherlands; 9 Department of Psychosis Studies, Institute of Psychiatry, King's Health Partners, King's College London, London, UK; 10 Department of Psychiatry, Brain Center Rudolf Magnus, Utrecht University Medical Centre, Utrecht, Utrecht, Netherlands; 11 ESRC Centre for Society and Mental Health and Social Epidemiology Research Group, King's College London, London, UK; 12 Health Service and Population Research Department, Centre for Epidemiology and Public Health, Institute of Psychiatry, Psychology & Neuroscience, King's College London, London, UK; 13 Department of Clinical, Neuro and Developmental Psychology, Vrije Universiteit, Amsterdam, North Holland, Netherlands; 14 Department of Psychosis Research, Parnassia Psychiatric Institute, The Hague, South Holland, Netherlands; 15 Department of Early Psychosis, Amsterdam UMC, Location AMC, University of Amsterdam, Amsterdam, North Holland, Netherlands; 16 Psychology Department, Institute of Psychiatry, Psychology and Neuroscience, King's College London, London, UK; 17 NIHR Biomedical Research Centre, South London and Maudsley NHS Foundation Trust, London, UK

**Keywords:** at-risk mental state, childhood abuse, transition, stress sensitization, ecological momentary assessment (EMA), experience sampling method (ESM)

## Abstract

**Aims:**

Childhood trauma is associated with an elevated risk for psychosis, but the psychological mechanisms involved remain largely unclear. This study aimed to investigate emotional and psychotic stress reactivity in daily life as a putative mechanism linking childhood trauma and clinical outcomes in individuals at ultra-high-risk (UHR) for psychosis.

**Methods:**

Experience sampling methodology was used to measure momentary stress, affect and psychotic experiences in the daily life of *N* = 79 UHR individuals in the EU-GEI High Risk Study. The Childhood Trauma Questionnaire was used to assess self-reported childhood trauma. Clinical outcomes were assessed at baseline, 1- and 2-year follow-up.

**Results:**

The association of stress with positive (*β* = −0.14, *p* = 0.010) and negative affect (*β* = 0.11, *p* = 0.020) was modified by transition status such that stress reactivity was greater in individuals who transitioned to psychosis. Moreover, the association of stress with negative affect (*β* = 0.06, *p* = 0.019) and psychotic experiences (*β* = 0.05, *p* = 0.037) was greater in individuals exposed to high *v*. low levels of childhood trauma. We also found evidence that decreased positive affect in response to stress was associated with reduced functioning at 1-year follow-up (*B* = 6.29, *p* = 0.034). In addition, there was evidence that the association of childhood trauma with poor functional outcomes was mediated by stress reactivity (e.g. indirect effect: *B* = −2.13, *p* = 0.026), but no evidence that stress reactivity mediated the association between childhood trauma and transition (e.g. indirect effect: *B* = 0.14, *p* = 0.506).

**Conclusions:**

Emotional and psychotic stress reactivity may be potential mechanisms linking childhood trauma with clinical outcomes in UHR individuals.

## Introduction

Meta-analytic evidence suggests that childhood trauma (i.e. potentially harmful experiences as sexual, physical and emotional abuse as well as physical and emotional neglect; Morgan and Fisher, 2007) increases transition risk in individuals at ultra-high-risk state for psychosis (UHR; Varese *et al*., 2012). Childhood trauma is associated with the persistence of psychotic symptoms in subclinical and clinical samples (Trotta *et al*., 2015; van Dam *et al*., 2015; Bailey *et al*., 2018). A UHR state is commonly based on three criteria (Fusar-Poli *et al*., 2015*a*; Fusar-Poli *et al*., 2016): attenuated psychotic symptoms, brief limited intermittent psychotic symptoms and genetic risk and deterioration syndrome. Within 2 years, 20% of UHR individuals have been reported to transition to psychosis (Fusar-Poli *et al*., 2016) and a considerable proportion experience comorbid anxiety or depression (Fusar-Poli *et al*., 2014). However, in recent years, declining transition rates have been reported and various reasons for this have been discussed (e.g. different clinical profiles, earlier referrals, more effective treatment; Yung *et al*., 2007; Hartmann *et al*., 2016; Nelson *et al*., 2016; Formica *et al*., 2020). Meta-analyses show that the majority of UHR individuals who do not transition to psychosis do not remit from UHR status within 2 years either, and show marked impairments in functioning (Simon *et al*., 2013; Fusar-Poli *et al*., 2015*b*). UHR individuals’ functional level is comparable to that reported in patients with social phobia or major depressive disorder, and closer to that observed in psychosis patients than in healthy controls (Fusar-Poli *et al*., 2015*b*). Hence, the persistence of symptoms and functioning are important outcomes.

Although it is well accepted that childhood trauma is associated with clinical outcomes, psychological mechanisms involved remain largely unclear. Current models of psychosis suggest that childhood trauma amplifies stress reactivity, comprising increased negative affect, decreased positive affect and increased psychotic experiences in response to minor daily stressors (Hammen *et al*., 2000; Kendler *et al*., 2004; Myin-Germeys and van Os, 2007; Collip *et al*., 2008; Morgan *et al*., 2010; Howes and Murray, 2014). Stress reactivity is thought to be a behavioural marker of stress sensitisation as a candidate mechanism underlying the association between childhood trauma and psychosis (Hammen *et al*., 2000; Myin-Germeys *et al*., 2001; Kendler *et al*., 2004; Wichers *et al*., 2009; Morgan *et al*., 2010, 2014; Bentall *et al*., 2014; Howes and Murray, 2014). There is evidence that stress reactivity in daily life is elevated in patients with psychosis, individuals with familial risk for psychosis, subclinical psychosis phenotypes and UHR individuals (Myin-Germeys *et al*., 2001, 2003; Lataster *et al*., 2009; Reininghaus *et al*., 2016*b*; van der Steen *et al*., 2017). Stress reactivity, measured with self-report questionnaires, has also been found to be associated with worse clinical outcomes in patients with first-episode psychosis (Conus *et al*., 2009). Furthermore, in adolescent service users, childhood trauma was associated with increased emotional and psychotic stress reactivity for individuals, who reported high *v*. low levels of trauma (Rauschenberg *et al*., 2017). This is consistent with other experience sampling studies showing elevated stress reactivity in patients of general practitioners, UHR individuals and in patients with psychosis, who have experienced childhood trauma (Glaser *et al*., 2006; Lardinois *et al*., 2011; Reininghaus *et al*., 2016*a*). Taken together, these findings suggest effect modification of stress reactivity by childhood trauma or, in other words, synergistic effects of trauma and stress reactivity, in those at-risk or with psychotic disorder (i.e. an interaction or synergistic model).

Furthermore, other possibilities of how childhood trauma and stress reactivity may combine with each other may be relevant (Schwartz and Susser, 2006; Morgan *et al*., 2014). Stress reactivity may take on the role of a mediator, such that childhood trauma may impact outcomes indirectly, via pathways through stress reactivity (i.e. a mediation model). In line with this, there is evidence from cross-sectional studies using self-report questionnaires in community samples that exposure to trauma in childhood may be linked to subclinical psychotic symptoms via stress reactivity (Gibson *et al*., 2014; Rössler *et al*., 2016). To increase complexity further, childhood trauma may both modify stress reactivity *and* connect with this putative mechanism along a causal pathway via mediation (Hafeman, 2008; Hafeman and Schwartz, 2009). In other words, exposure to trauma may interact with, and be predictive of, stress reactivity in pathways to psychosis (i.e. a mediated synergy model). To our knowledge, only one study to date has investigated both effect modification and mediation in the same analyses in relation to psychosis, suggesting that childhood and adult disadvantage may combine in complex ways (Morgan *et al*., 2014). Although stress reactivity may be an important putative risk mechanism, no study to date has investigated whether stress reactivity in UHR individuals’ daily life is greater in those exposed to high levels of childhood trauma, as well as its predictive value for clinical outcomes (Reininghaus *et al*., 2016*a*, 2016*b*). Therefore, the aim of the current study was to investigate the interplay of exposure to childhood trauma and stress reactivity as a candidate mechanism in predicting clinical outcomes in UHR individuals at 1- and 2-year follow-up using experience sampling data. We tested, in light of the theoretical models outlined above, the following hypotheses (see online Supplementary Fig. S1):

(H1) An increase in momentary stress is associated with increased negative affect, decreased positive affect and increased psychotic experiences.
(H2) The magnitude of associations between momentary stress and negative affect, positive affect and psychotic experiences is modified by childhood trauma, such that these associations are greater in individuals exposed to high *v*. low levels of childhood trauma (i.e. an effect modification or interaction model).
(H3) Stress reactivity (measured at baseline) predicts illness severity, functioning and symptom burden at 1- and 2-year follow-up.
(H4) Childhood trauma (measured at baseline) predicts illness severity, functioning and symptom burden at 1- and 2-year follow-up. The effects of childhood trauma will be mediated via pathways through stress reactivity (i.e. a mediation model).

In exploratory analyses, we further aimed to investigate whether (i) the magnitude of associations between momentary stress and negative affect, positive affect and psychotic experiences is modified by transition status, and (ii) the effect of childhood trauma on transition status will be mediated via pathways through stress reactivity (i.e. a mediation model).

## Methods

### Sample

The sample comprises UHR individuals from London (UK), Melbourne (Australia) and Amsterdam/The Hague (the Netherlands) recruited as part of the EU-GEI High Risk Study (European Network of National Networks studying Gene–Environment Interactions in Schizophrenia, 2014), a naturalistic prospective multicentre study that aimed to identify the interactive genetic, clinical and environmental determinants of schizophrenia. For the UK, participants were recruited from Outreach and Support in South London (OASIS), a clinical service for UHR individuals provided by the South London and Maudsley NHS Foundation Trust (Fusar-Poli *et al*., 2013), the West London Mental Health NHS Trust (WLMHT), and a community survey of General Practitioner practices (Reininghaus *et al*., 2016*a*). In Melbourne, participants were recruited from the Personal Assessment and Crisis Evaluation (PACE) clinic, a clinical arm of Orygen Youth Health, whose catchment area includes the north-western metropolitan region of Melbourne. Dutch participants were recruited from the Early Detection for Psychosis clinics of Parnassia, The Hague, and Amsterdam UMC. All centres provide assessments and specialised clinical services for people with UHR.

UHR individuals, aged 15–35 years, were eligible to participate if they met at least one of the UHR criteria as defined by the Comprehensive Assessment of At Risk Mental State (CAARMS; Yung *et al*., 2005): (1) attenuated psychotic symptoms: the presence of subthreshold positive psychotic symptoms for at least 1 month during the past year, (2) brief limited intermittent psychotic symptoms: an episode of frank psychotic symptoms that have resolved in less than 1 week without receiving treatment and (3) vulnerability: a first-degree relative with a psychotic disorder or diagnosed with schizotypal personality disorder in combination with a significant drop in functioning or chronic low functioning during at least 1 month in the previous year. Exclusion criteria were: (1) the presence of a current or past psychotic disorder, (2) symptoms relevant for inclusion are explained by a medical disorder or drugs/alcohol dependency and (3) IQ < 60.

### Data collection

#### Experience sampling method (ESM) measures

Momentary stress, affect and psychotic experiences were assessed using the ESM (Myin-Germeys *et al*., 2001; Palmier-Claus *et al*., 2012), a structured diary method with high ecological validity, in which subjects are asked to report their thoughts, feelings and symptoms in daily life (Shiffman *et al*., 2008; Myin-Germeys *et al*., 2009; Palmier-Claus *et al*., 2011). At baseline, participants used a dedicated digital device for data collection (the Psymate^®^, www.psymate.eu/). The target constructs (i.e. stress, affect and psychotic experiences) show high and continuous variation over time. To obtain a representative sample of participants’ experiences in daily life and to capture relevant variation in these target constructs with high resolution, a time-contingent sampling design with a blocked random schedule and a high-sampling frequency was used for ESM data collection, i.e. ten times a day for six consecutive days at random moments within set blocks of time (Shiffman *et al*., 2008; Myin-Germeys *et al*., 2018). In line with previous literature, data were included if ⩾20 valid responses were provided over the assessment period (Myin-Germeys *et al*., 2001, 2005; Delespaul *et al*., 2002; Corcoran *et al*., 2006; Bentall *et al*., 2008, 2009; Freeman *et al*., 2013; Reininghaus *et al*., 2016*b*). A detailed description of the ESM procedure and measures is provided in online Supplementary material 2.

#### Childhood trauma

Childhood trauma was assessed using the short form of the Childhood Trauma Questionnaire (CTQ), an established 25-item self-report measure enquiring about traumatic experiences during childhood (for detailed information see online Supplementary material 2; Bernstein *et al*., 1997, 2003; Bernstein and Fink, 1998; Scher *et al*., 2001; Wingenfeld *et al*., 2010).

#### Clinical outcomes

Clinical outcomes were assessed at baseline, 1- and 2-year follow-up. As the time points for follow-up assessments varied, the data closest to 1 and 2 years after baseline were selected as follow-up data. Illness severity was assessed using the Clinical Global Impression Scale (CGI; Guy, 1976). The level of functioning was assessed using the Global Assessment of Functioning Scale (GAF; American Psychiatric Association, 2002). Symptoms were assessed using the unusual thought content, perceptual abnormalities, anxiety and tolerance to normal stress subscales of the CAARMS (Yung *et al*., 2005). To ensure data quality, extensive training was provided (see online Supplementary material 3).

### Statistical analysis

As ESM data have a multilevel structure with multiple observations (level-1) nested within participants (level-2), the ‘mixed’ command in Stata 15 was used to fit two-level, linear mixed models (StataCorp, 2017). Continuous variables of momentary stress, affect, psychotic experiences and childhood trauma were *z*-standardised for interpreting significant interaction terms. First, we included the composite stress measure as an independent variable and negative affect, positive affect and psychotic experiences as outcome variables (H1). Second, we added two-way interaction terms for stress × childhood trauma to examine whether the associations between momentary stress, negative affect, positive affect and psychotic experiences were modified by childhood trauma (H2). The hypothesis that the associations of momentary stress with affect and psychotic experiences were greater in individuals exposed to high *v*. low levels of childhood trauma (±1 s.d. of standardised CTQ scores, mean = 0, s.d. = 1) was tested by using the ‘testparm’ command for computing Wald tests to assess statistical significance of two-way interaction terms and the ‘lincom’ command to compute linear combinations of coefficients (Aiken and West, 1991; Cohen *et al*., 2003). Third, we used the ‘predict’ option to obtain fitted values of psychotic experiences and affect predicted by the composite stress measure. We used linear regression analysis to investigate whether these fitted values representing stress reactivity predicted illness severity, level of functioning and symptom burden at follow-up, while controlling for baseline values (H3). Finally, we performed mediation analysis using the ‘gsem’ command to investigate whether the effects of childhood trauma on illness severity, level of functioning and symptom burden were mediated by stress reactivity (H4). The total effect of childhood trauma on clinical outcomes was apportioned into a direct effect and an indirect effect through stress reactivity. The indirect effect was computed using the product of coefficients strategy. The indirect and the total effect were computed and tested on significance using the ‘nlcom’ command.

Restricted maximum-likelihood (H1 and H2) or maximum-likelihood estimation (H3 and H4) were applied, allowing for the use of all available data under the relatively unrestrictive assumption that data are missing at random and if all variables associated with missing values are included in the model (Little and Rubin, 1987; Mallinckrodt *et al*., 2001). Following previous studies (Reininghaus *et al*., 2016*a*, 2016*b*; Rauschenberg *et al*., 2017; Hermans *et al*., 2020), all analyses were adjusted for age, gender, ethnicity and centre as these are known as *a priori* confounders (based on evidence on the basic epidemiology of psychosis). To control for confounding of findings by comorbid disorders, all analyses were controlled for comorbid major depressive and anxiety disorders. In addition, analyses for testing H3 and H4 were controlled for time to follow-up to account for variation in time to follow-up. Unadjusted analyses and sensitivity analyses in a restricted sample assessed in a ±6 month time interval around the expected follow-up time points are displayed in online Supplementary materials 4–6.

## Results

### Basic sample and clinical characteristics

A total of 108 participants were assessed with the ESM during the study period. Of these, 79 participants completed ESM assessment with ⩾20 valid responses (i.e. 73.1% of 108; valid responses: *M* = 38, range 20–57). Assessment of clinical outcomes was completed for 48 participants at 1-year follow-up (61% of the full sample; months away from optimal 1-year follow-up time point: median = 0.5, range −8.7 to 4.6) and 36 participants at 2-year follow-up (46% of the full sample; months away from optimal 2-year follow-up time point: median = 0.5, range −5.6 to 22.6). Nine individuals (11%) transitioned to psychosis by the final follow-up time point. Participants were on average 23 years old (s.d. = 4.93) and 56% were women. The majority (67%) of the sample was white, followed by 15% with black ethnicity. Seventy-six percent of the participants were diagnosed with a comorbid axis I disorder. Comparing the current study's participants to individuals included in the EU GEI High-Risk study, for whom ESM data were not collected (*N* = 266), there were no differences in demographics (age: *t* = −1.33, *p* = 0.185; gender: *χ*^2^ = 3.58, *p* = 0.059; ethnicity: *χ*^2^ = 6.53, *p* = 0.258) or overall prevalence of comorbid disorders (*χ*^2^ = 1.82, *p* = 0.177). However, the current sample showed higher levels of childhood trauma (*t* = −2.59, *p* = 0.010), a higher prevalence of specific phobias (*χ*^2^ = 4.86, *p* = 0.027) and a lower prevalence of major depressive disorder (*χ*^2^ = 4.67, *p* = 0.031) compared to participants, for whom ESM data were not collected (see Table 1).

**Table 1.** Basic sample and clinical characteristics

	ESM sample			No ESM sample	Comparison ESM *v*. no ESM
	Baseline	1-year follow-up	2-year follow-up	Baseline	Baseline
Sample size *N*	79	48	36	266	
Age at baseline (years), mean (s.d.)	23.0 (4.93)	23.6 (5.24)	23.81 (5.18)	22.2 (4.82)	*t* = −1.33, *p* = 0.185
Gender *N* (%)					*χ*^2^ = 3.58, *p* = 0.059
Male	35 (44%)	22 (46%)	16 (44%)	150 (56%)	
Female	44 (56%)	26 (54%)	20 (56%)	116 (44%)	
Ethnicity *N* (%)					*χ*^2^ = 6.53, *p* = 0.258
White	53 (67%)	33 (69%)	27 (75%)	193 (73%)	
Black	12 (15%)	9 (19%)	5 (14%)	22 (8%)	
Other	14 (18%)	6 (13%)	4 (11%)	50 (19%)	
Comorbidity at baseline *N* (%)	60 (76%)	37 (77%)	28 (78%)	220 (83%)	*χ*^2^ = 1.82, *p* = 0.177
Major depressive disorder *N* (%)	29 (37%)	14 (31%)	11 (31%)	123 (51%)	*χ*^2^ = 4.67, *p* = 0.031
Current depressive episode *N* (%)	22 (28%)	11 (24%)	8 (22%)	88 (35%)	*χ*^2^ = 1.26, *p* = 0.262
Bipolar disorder *N* (%)	7 (9%)	4 (9%)	5 (14%)	17 (6%)	*χ*^2^ = 0.57, *p* = 0.449
Any anxiety disorder *N* (%)	42 (53%)	26 (57%)	17 (47%)	117 (44%)	*χ*^2^ = 2.06, *p* = 0.151
Panic disorder *N* (%)	19 (24%)	12 (27%)	6 (17%)	52 (21%)	*χ*^2^ = 0.30, *p* = 0.584
Panic disorder + agoraphobia *N* (%)	6 (8%)	4 (9%)	1 (3%)	25 (11%)	*χ*^2^ = 0.46, *p* = 0.496
Agoraphobia only *N* (%)	2 (3%)	0	0	4 (2%)	*χ*^2^ = 0.26, *p* = 0.607
Social phobia *N* (%)	19 (24%)	14 (30%)	9 (25%)	42 (17%)	*χ*^2^ = 1.87, *p* = 0.172
Specific phobia *N* (%)	14 (18%)	9 (20%)	5 (14%)	22 (9%)	*χ*^2^ = 4.86, *p* = 0.027
Generalised anxiety disorder *N* (%)	11 (14%)	7 (15%)	5 (14%)	26 (11%)	*χ*^2^ = 0.67, *p* = 0.413
Not otherwise specified anxiety disorder *N* (%)	3 (4%)	1 (2%)	0	14 (6%)	*χ*^2^ = 0.49, *p* = 0.485
Obsessive-compulsive disorder *N* (%)	3 (4%)	2 (4%)	3 (9%)	26 (12%)	*χ*^2^ = 3.41, *p* = 0.065
Posttraumatic stress disorder *N* (%)	11 (14%)	4 (9%)	0	23 (6%)	*χ*^2^ = 1.40, *p* = 0.237
Any eating disorder *N* (%)	10 (13%)	7 (15%)	6 (17%)	22 (8%)	*χ*^2^ = 1.39, *p* = 0.238
Anorexia nervosa *N* (%)	5 (6%)	3 (7%)	3 (8%)	10 (4%)	*χ*^2^ = 0.69, *p* = 0.408
Bulimia nervosa *N* (%)	5 (6%)	3 (7%)	2 (6%)	10 (4%)	*χ*^2^ = 0.66, *p* = 0.417
Binge eating disorder *N* (%)	1 (1%)	1 (2%)	1 (3%)	6 (3%)	*χ*^2^ = 0.44, *p* = 0.508
Any somatoform disorder *N* (%)	2 (3%)	1 (2%)	1 (3%)	9 (3%)	*χ*^2^ = 0.14, *p* = 0.705
Somatisation disorder *N* (%)	1 (1%)	0	0	4 (2%)	*χ*^2^ = 0.06, *p* = 0.812
Chronic pain *N* (%)	1 (1%)	0	0	1 (<1%)	*χ*^2^ = 0.70, *p* = 0.403
Hypochondriasis *N* (%)	1 (1%)	1 (2%)	1 (3%)	4 (2%)	*χ*^2^ = 0.07, *p* = 0.789
Body dysmorphic disorder *N* (%)	0	0	0	2 (1%)	*χ*^2^ = 0.67, *p* = 0.412
Childhood trauma questionnaire total score at baseline, mean (s.d.)	51.54 (17.00)	50.13 (15.60)	47.33 (13.31)	46.23 (14.97)	*t* = −2.59, *p* = 0.010
Clinical global impression scale illness severity, mean (s.d.)	3.57 (1.21)	3.15 (1.32)	2.89 (1.26)	3.60 (1.09)	*t* = 0.21, *p* = 0.831
Global assessment of functioning					
Disability, mean (s.d.)	56.27 (13.00)	58.92 (13.41)	63.78 (13.62)	55.36 (12.20)	*t* = −0.57, *p* = 0.572
Comprehensive assessment of at risk mental states					
Unusual thought content, mean (s.d.)	2.89 (1.77)	2.13 (1.94)	1.62 (1.95)	2.68 (1.85)	*t* = −0.88, *p* = 0.378
Perceptual abnormalities, mean (s.d.)	3.08 (1.65)	2.42 (1.69)	1.85 (1.84)	2.84 (1.67)	*t* = −1.13, *p* = 0.261
Anxiety, mean (s.d.)	3.29 (1.29)	2.89 (1.45)	2.59 (1.83)	2.99 (1.68)	*t* = −1.47, *p* = 0.144
Tolerance to normal stress, mean (s.d.)	2.09 (1.85)	1.04 (1.57)	1.00 (1.61)	2.13 (1.77)	*t* = 0.19, *p* = 0.850

*Note*. ESM, experience sampling method; *N*, sample size, s.d., standard deviation. Comorbidity: participants were diagnosed with a comorbid disorder, if classification criteria were fulfilled. Thus, one participant can be diagnosed with multiple comorbid disorders.

### Association between momentary stress, affect and psychotic experiences (H1)

Momentary stress was associated with small to moderate increases in negative affect (*β* = 0.31, 95% confidence interval (CI) 0.27 to 0.36, *p* < 0.001) and psychotic experiences (*β* = 0.16, 95% CI 0.13 to 0.20, *p* < 0.001) as well as with a moderate decrease in positive affect (*β* = −0.38, 95% CI −0.43 to −0.34, *p* < 0.001).

### Association between momentary stress, affect and psychotic experiences by childhood trauma (H2)

Childhood trauma modified the associations of momentary stress with negative affect (stress × childhood trauma: *β* = 0.03, 95% CI 0.00 to 0.06, *p* = 0.019) and psychotic experiences (stress × childhood trauma: *β* = 0.02, 95% CI 0.00 to 0.05, *p* = 0.044, see Table 2). These associations were greater in individuals with higher levels of childhood trauma (outcome negative affect: high *v*. low childhood trauma: *β* = 0.06, 95% CI 0.01 to 0.11, *p* = 0.019; outcome psychotic experiences: high *v*. low childhood trauma: *β* = 0.05, 95% CI 0.00 to 0.09, *p* = 0.044). Furthermore, we found a non-significant indication that childhood trauma modified the association between momentary stress and positive affect (stress × childhood trauma: *β* = 0.03, 95% CI 0.00 to 0.06, *p* = 0.081).

**Table 2.** Modification of the association between momentary stress and affect/psychotic experiences by childhood trauma

Effect modification by childhood trauma				
	*β*	95% CI	s.e.	*p*
*Outcome: negative affect*				
Stress	0.31	0.28 to 0.34	0.01	<0.001
Childhood trauma	0.23	0.08 to 0.38	0.08	0.003
Stress × childhood trauma	0.03	0.00 to 0.06	0.01	0.019
High childhood trauma	0.34	0.31 to 0.37	0.02	<0.001
Low childhood trauma	0.28	0.24 to 0.32	0.02	<0.001
High *v*. low childhood trauma	0.06	0.01 to 0.11	0.03	0.019
*Outcome: positive affect*				
Stress	−0.39	−0.42 to −0.36	0.02	<0.001
Childhood trauma	−0.07	−0.21 to 0.07	0.07	0.311
Stress × childhood trauma	0.03	0.00 to 0.06	0.02	0.081
*Outcome: psychotic experiences*				
Stress	0.15	0.13 to 0.17	0.01	<0.001
Childhood trauma	0.28	0.12 to 0.44	0.08	0.001
Stress × childhood trauma	0.02	0.00 to 0.05	0.01	0.044
High childhood trauma	0.17	0.14 to 0.20	0.02	<0.001
Low childhood trauma	0.13	0.09 to 0.16	0.02	<0.001
High *v*. low childhood trauma	0.05	0.00 to 0.09	0.02	0.044

*Note*: Results adjusted for age, gender, ethnicity, centre, comorbid major depressive and anxiety disorders. Childhood trauma assessed with the CTQ. 95% CI, 95% confidence interval, s.e., standard error.

### Stress reactivity and clinical outcomes at follow-up (H3)

Decreased positive affect in response to stress was associated with higher illness severity (*B* = −0.51, 95% CI −0.97 to −0.06, *p* = 0.028) and lower level of functioning (*B* = 7.92, 95% CI 1.39 to 14.45, *p* = 0.019) at 1-year follow-up (see Table 3). In addition, the level of functioning at 2-year follow-up was predicted by psychotic stress reactivity (*B* = 11.62, 95% CI 1.70 to 21.54, *p* = 0.024).^1^ Increased negative affect in response to stress predicted unusual thought content at 2-year follow-up (*B* = 1.74, 95% CI 0.36 to 3.11, *p* = 0.016). Moreover, perceptual abnormalities at 1-year follow-up were predicted by emotional (negative affect: *B* = 1.24, 95% CI 0.54 to 1.93, *p* = 0.001; positive affect: *B* = −1.03, 95% CI −1.81 to −0.25, *p* = 0.011) and psychotic stress reactivity (*B* = 1.06, 95% CI 0.29 to 1.83, *p* = 0.009). There was no evidence that emotional or psychotic stress reactivity predicted anxiety or tolerance to normal stress.

**Table 3.** Clinical outcomes at 1- and 2-year follow-up predicted by emotional and psychotic stress reactivity at baseline and clinical outcome at baseline

	Clinical outcomes							
	Illness severity (CGI)				Level of functioning disability (GAF)			
	1-year follow-up (*N* = 46)		2-year follow-up (*N* = 35)		1-year follow-up (*N* = 47)		2-year follow-up (*N* = 35)	
	*B* (95% CI)	*p*	*B* (95% CI)	*p*	*B* (95% CI)	*p*	*B* (95% CI)	*p*
Predictor: emotional reactivity (increased negative affect in response to stress)								
Outcome at baseline	0.62 (0.35 to 0.89)	<0.001	0.27 (−0.24 to 0.77)	0.290	0.35 (0.01 to 0.70)	0.047	0.51 (0.02 to 1.00)	0.041
Emotional reactivity	0.38 (−0.15 to 0.91)	0.156	0.02 (−0.92 to 0.96)	0.963	−5.17 (−12.54 to 2.20)	0.163	1.31 (−8.38 to 11.01)	0.782
Predictor: emotional reactivity (decreased positive affect in response to stress)								
Outcome at baseline	0.60 (0.35 to 0.86)	<0.001	0.16 (−0.34 to 0.66)	0.520	0.34 (0.01 to 0.66)	0.044	0.50 (0.01 to 0.99)	0.046
Emotional reactivity	−0.51 (−0.97 to −0.06)	0.028	−0.50 (−1.44 to 0.44)	0.282	7.92 (1.39 to 14.45)	0.019	−0.17 (−9.48 to 9.15)	0.971
Predictor: psychotic reactivity (increased psychotic experiences in response to stress)								
Outcome at baseline	0.70 (0.42 to 0.98)	<0.001	0.38 (−0.12 to 0.88)	0.129	0.41 (0.07 to 0.76)	0.021	0.54 (0.11 to 0.98)	0.016
Psychotic reactivity	−0.04 (−0.64 to 0.55)	0.863	−0.58 (1.68 to 0.51)	0.283	−1.59 (−9.08 to 5.90)	0.669	11.62 (1.70 to 21.54)	0.024
	Unusual thought content (CAARMS)				Perceptual abnormalities (CAARMS)			
	1-year follow-up (*N* = 43)		2-year follow-up (*N* = 33)		1-year follow-up (*N* = 43)		2-year follow-up (*N* = 32)	
	*B* (95% CI)	*p*	*B* (95% CI)	*p*	*B* (95% CI)	*p*	*B* (95% CI)	*p*
Predictor: emotional reactivity (increased negative affect in response to stress)								
Outcome at baseline	0.43 (0.09 to 0.78)	0.016	−0.12 (−0.58 to 0.34)	0.595	0.40 (0.16 to 0.64)	0.002	0.37 (−0.13 to 0.87)	0.142
Emotional reactivity	0.47 (−0.50 to 1.45)	0.331	1.74 (0.36 to 3.11)	0.016	1.24 (0.54 to 1.93)	0.001	−0.11 (−1.55 to 1.34)	0.878
Predictor: emotional reactivity (decreased positive affect in response to stress)								
Outcome at baseline	0.43 (0.08 to 0.77)	0.016	−0.08 (−0.58 to 0.41)	0.727	0.45 (0.19 to 0.71)	0.001	0.42 (−0.08 to 0.92)	0.093
Emotional reactivity	−0.71 (−1.71 to 0.30)	0.162	−1.09 (−2.44 to 0.25)	0.105	−1.03 (−1.81 to −0.25)	0.011	−0.51 (−1.79 to 0.77)	0.416
Predictor: psychotic reactivity (increased psychotic experiences in response to stress)								
Outcome at baseline	0.42 (0.05 to 0.79)	0.029	−0.14 (−0.65 to 0.38)	0.592	0.33 (0.06 to 0.59)	0.018	0.38 (−0.11 to 0.86)	0.121
Psychotic reactivity	0.27 (−0.77 to 1.32)	0.599	1.26 (−0.28 to 2.81)	0.103	1.06 (0.29 to 1.83)	0.009	0.51 (−0.90 to 1.91)	0.460
	Anxiety (CAARMS)				Tolerance to normal stress (CAARMS)			
	1-year follow-up (*N* = 43)		2-year follow-up (*N* = 33)		1-year follow-up (*N* = 43)		2-year follow-up (*N* = 33)	
	*B* (95% CI)	*p*	*B* (95% CI)	*p*	*B* (95% CI)	*p*	*B* (95% CI)	*p*
Predictor: emotional reactivity (increased negative affect in response to stress)								
Outcome at baseline	0.29 (−0.17 to 0.75)	0.207	0.54 (−0.54 to 1.62)	0.312	0.35 (0.07 to 0.63)	0.016	0.25 (−0.13 to 0.63)	0.191
Emotional reactivity	0.14 (−0.61 to 0.89)	0.699	−0.64 (−2.19 to 0.91)	0.402	−0.10 (−0.94 to 0.74)	0.816	−0.48 (−1.77 to 0.81)	0.447
Predictor: emotional reactivity (decreased positive affect in response to stress)								
Outcome at baseline	0.23 (−0.19 to 0.64)	0.268	0.37 (−0.65 to 1.40)	0.455	0.34 (0.07 to 0.62)	0.017	0.20 (−0.16 to 0.57)	0.265
Emotional reactivity	−0.68 (−1.39 to 0.02)	0.057	0.00 (−1.32 to 1.32)	0.997	0.22 (−0.65 to 1.09)	0.609	0.10 (−1.01 to 1.21)	0.850
Predictor: psychotic reactivity (increased psychotic experiences in response to stress)								
Outcome at baseline	0.31 (−0.13 to 0.75)	0.156	0.54 (−0.45 to 1.53)	0.272	0.35 (0.07 to 0.63)	0.016	0.25 (−0.14 to 0.64)	0.197
Psychotic reactivity	0.08 (−0.64 to 0.80)	0.824	−1.07 (−2.49 to 0.35)	0.131	−0.10 (−0.95 to 0.75)	0.815	−0.44 (−1.75 to 0.87)	0.489

*Note*: Results adjusted for age, gender, ethnicity, centre, comorbid major depressive and anxiety disorders and time to follow-up. Illness severity assessed with the Clinical Global Impression Scale (CGI). Level of functioning assessed with the Global Assessment of Functioning Scale (GAF). *N*, sample size; 95% CI, 95% confidence interval.

### Emotional and psychotic stress reactivity as mediators of the association between childhood trauma and clinical outcomes (H4)

Table 4 shows findings on total, direct and indirect effects of childhood trauma and stress reactivity on clinical outcomes at follow-up. Increased negative affect in response to stress mediated the association of childhood trauma and illness severity at 1-year follow-up (indirect effect: *B* = 0.20, 95% CI 0.02 to 0.38, *p* = 0.033). We found no evidence that emotional and psychotic stress reactivity mediated the association of childhood trauma and level of functioning. The association of childhood trauma and unusual thought content at 2-year follow-up was mediated by increased negative affect in response to stress (*B* = 0.42, 95% CI 0.04 to 0.80, *p* = 0.030). In addition, the association of childhood trauma and perceptual abnormalities at 1-year follow-up was mediated by increased negative affect (indirect effect: *B* = 0.39, 95% CI 0.09 to 0.69, *p* = 0.011) and psychotic experiences in response to stress (indirect effect: *B* = 0.44, 95% CI 0.13 to 0.75, *p* = 0.005). High levels of childhood trauma were associated with more intense reactivity in the form of a stronger increase of negative affect and psychotic experiences in response to stress, which, in turn, was associated with higher illness severity, unusual thought content and perceptual abnormalities at follow-up. We found no evidence for direct effects of childhood trauma on anxiety and tolerance to normal stress and no mediation via stress reactivity.

**Table 4.** Emotional and psychotic stress reactivity as mediators of the association of childhood trauma and clinical outcomes

	Clinical outcomes							
	Illness severity (CGI)				Level of functioning disability (GAF)			
	1-year follow-up (*N* = 47)		2-year follow-up (*N* = 36)		1-year follow-up (*N* = 47)		2-year follow-up (*N* = 35)	
	*B* (95% CI)	*p*	*B* (95% CI)	*p*	*B* (95% CI)	*p*	*B* (95% CI)	*p*
Mediator: emotional reactivity (increased negative affect in response to stress)								
Total effect	0.39 (0.06 to 0.72)	0.022	−0.43 (−0.90 to 0.04)	0.074	−3.48 (−7.47 to 0.51)	0.087	3.55 (−1.84 to 8.95)	0.197
Direct effect	0.19 (−0.12 to 0.51)	0.224	−0.57 (−1.07 to −0.06)	0.027	−1.82 (−5.79 to 2.16)	0.371	4.27 (−1.59 to 10.14)	0.153
Indirect effect	0.20 (0.02 to 0.38)	0.033	0.14 (−0.06 to 0.33)	0.170	−1.67 (−3.65 to 0.32)	0.100	−0.72 (−2.98 to 1.54)	0.531
Mediator: emotional reactivity (decreased positive affect in response to stress)								
Total effect	0.33 (0.02 to 0.65)	0.038	−0.40 (−0.86 to 0.06)	0.089	−3.38 (−7.20 to 0.45)	0.083	3.44 (−1.96 to 8.83)	0.212
Direct effect	0.24 (−0.05 to 0.54)	0.110	−0.48 (−0.93 to −0.03)	0.037	−2.30 (−5.93 to 1.32)	0.213	3.69 (−1.73 to 9.11)	0.182
Indirect effect	0.09 (−0.03 to 0.22)	0.142	0.08 (−0.04 to 0.20)	0.188	−1.07 (−2.47 to 0.33)	0.133	−0.25 (−1.19 to 0.68)	0.592
Mediator: psychotic reactivity (increased psychotic experiences in response to stress)								
Total effect	0.36 (0.02 to 0.69)	0.039	−0.42 (−0.90 to 0.07)	0.091	−2.96 (−6.97 to 1.05)	0.148	4.26 (−1.10 to 9.62)	0.119
Direct effect	0.22 (−0.12 to 0.55)	0.202	−0.42 (−0.93 to 0.09)	0.103	−2.72 (−6.92 to 1.48)	0.205	1.58 (−3.86 to 7.03)	0.569
Indirect effect	0.14 (−0.04 to 0.32)	0.132	0.01 (−0.23 to 0.24)	0.949	0.24 (−2.32 to 1.84)	0.821	2.67 (−0.28 to 5.63)	0.076
	Unusual thought content (CAARMS)				Perceptual abnormalities (CAARMS)			
	1-year follow-up (*N* = 43)		2-year follow-up (*N* = 33)		1-year follow-up (*N* = 43)		2-year follow-up (*N* = 32)	
	*B* (95% CI)	*p*	*B* (95% CI)	*p*	*B* (95% CI)	*p*	*B* (95% CI)	*p*
Mediator: emotional reactivity (increased negative affect in response to stress)								
Total effect	−0.21 (−0.77 to 0.35)	0.469	0.41 (−0.36 to 1.18)	0.297	0.03 (−0.44 to 0.51)	0.886	−0.08 (−0.87 to 0.72)	0.852
Direct effect	−0.42 (−1.02 to 0.18)	0.166	−0.01 (−0.78 to 0.76)	0.983	−0.36 (0.81 to 0.09)	0.120	0.01 (−0.83 to 0.85)	0.986
Indirect effect	0.22 (−0.06 to 0.50)	0.125	0.42 (0.04 to 0.80)	0.030	0.39 (0.09 to 0.69)	0.011	−0.08 (−0.39 to 0.23)	0.598
Mediator: emotional reactivity (decreased positive affect in response to stress)								
Total effect	−0.15 (−0.71 to 0.41)	0.598	0.37 (−0.41 to 1.16)	0.350	0.10 (−0.37 to 0.58)	0.667	−0.06 (−0.86 to 0.73)	0.874
Direct effect	−0.25 (−0.79 to 0.30)	0.374	0.26 (−0.52 to 1.04)	0.511	0.00 (−0.46 to 0.45)	0.990	−0.10 (−0.90 to 0.71)	0.812
Indirect effect	0.10 (−0.06 to 0.25)	0.226	0.11 (−0.07 to 0.29)	0.215	0.11 (−0.05 to 0.26)	0.179	0.03 (−0.09 to 0.16)	0.604
Mediator: psychotic reactivity (increased psychotic experiences in response to stress)								
Total effect	−0.20 (−0.76 to 0.36)	0.490	0.48 (−0.32 to 1.28)	0.241	0.05 (−0.42 to 0.52)	0.825	0.00 (−0.81 to 0.80)	0.993
Direct effect	−0.47 (−1.07 to 0.13)	0.126	0.19 (−0.61 to 0.99)	0.642	−0.39 (−0.84 to 0.07)	0.095	−0.16 (−0.97 to 0.65)	0.701
Indirect effect	0.27 (−0.03 to 0.58)	0.080	0.29 (−0.06 to 0.64)	0.105	0.44 (0.13 to 0.75)	0.005	0.16 (−0.18 to 0.49)	0.363
	Anxiety (CAARMS)				Tolerance to normal stress (CAARMS)			
	1-year follow-up (*N* = 43)		2-year follow-up (*N* = 33)		1-year follow-up (*N* = 43)		2-year follow-up (*N* = 33)	
	*B* (95% CI)	*p*	*B* (95% CI)	*p*	*B* (95% CI)	*p*	*B* (95% CI)	*p*
Mediator: emotional reactivity (increased negative affect in response to stress)								
Total effect	−0.19 (−0.56 to 0.18)	0.315	−0.46 (−1.25 to 0.33)	0.255	−0.15 (−0.62 to 0.32)	0.531	−0.01 (−0.68 to 0.67)	0.982
Direct effect	−0.34 (−0.75 to 0.08)	0.111	−0.42 (−1.25 to 0.41)	0.326	−0.19 (−0.72 to 0.33)	0.464	0.04 (−0.67 to 0.75)	0.913
Indirect effect	0.14 (−0.04 to 0.32)	0.137	−0.04 (−0.34 to 0.26)	0.791	0.04 (−0.17 to 0.26)	0.688	−0.05 (−0.31 to 0.21)	0.719
Mediator: emotional reactivity (decreased positive affect in response to stress)								
Total effect	−0.14 (−0.50 to 0.22)	0.453	−0.45 (−1.24 to 0.34)	0.260	−0.16 (−0.64 to 0.31)	0.502	0.00 (−0.68 to 0.67)	0.994
Direct effect	−0.23 (−0.57 to 0.11)	0.187	−0.46 (−1.26 to 0.33)	0.253	−0.14 (−0.61 to 0.34)	0.576	0.00 (−0.69 to 0.68)	0.989
Indirect effect	0.09 (−0.04 to 0.22)	0.162	0.01 (−0.11 to 0.13)	0.855	−0.03 (−0.12 to 0.07)	0.577	0.00 (−0.10 to 0.10)	0.964
Mediator: psychotic reactivity (increased psychotic experiences in response to stress)								
Total effect	−0.18 (−0.55 to 0.19)	0.332	−0.54 (−1.33 to 0.24)	0.176	−0.15 (−0.62 to 0.32)	0.536	−0.01 (−0.70 to 0.67)	0.968
Direct effect	−0.30 (−0.71 to 0.11)	0.152	−0.32 (−1.11 to 0.47)	0.425	−0.22 (−0.75 to 0.31)	0.413	0.01 (−0.68 to 0.71)	0.968
Indirect effect	0.11 (−0.08 to 0.31)	0.241	−0.22 (−0.55 to 0.11)	0.193	0.07 (−0.17 to 0.31)	0.562	−0.03 (−0.30 to 0.25)	0.841

*Note*: Results adjusted for age, gender, ethnicity, centre, comorbid major depressive and anxiety disorders and time to follow-up. Childhood trauma assessed with the CTQ. Illness severity assessed with the Clinical Global Impression Scale (CGI). Level of functioning assessed with the Global Assessment of Functioning Scale (GAF). Unusual thought content, perceptual abnormalities, anxiety and tolerance to normal stress assessed with the Comprehensive Assessment of At Risk Mental State (CAARMS). *N*, sample size, 95% CI, 95% confidence interval.

In exploratory analyses, there was no evidence for a direct effect of childhood trauma on transition status and no mediation via stress reactivity (see online Supplementary material 7).

## Discussion

### Main findings

Using an experience sampling design, we found strong evidence that minor daily stressors were associated with emotional and psychotic stress reactivity in UHR individuals (H1). Childhood trauma modified the effect of daily stressors on negative affect and psychotic experiences, with more intense psychotic experiences and stronger increases in negative affect for individuals exposed to high levels of childhood trauma (H2). In addition, we found some evidence to suggest stress reactivity predicts clinical outcomes at follow-up (H3). Finally, there was partial evidence that stress reactivity mediates the association of childhood trauma and clinical outcomes (H4).

### Methodological considerations/limitations

The reported findings should be interpreted in light of several methodological considerations. First, childhood trauma was measured with a retrospective self-report questionnaire. A common concern about retrospective self-report is that recall bias and cognitive distortions might lead to invalid ratings (Dill *et al*., 1991; Saykin *et al*., 1991; Morgan and Fisher, 2007; Susser and Widom, 2012; Colman *et al*., 2016). However, good reliability and validity for these measures have been reported in individuals with psychosis (Fisher *et al*., 2011). Similar levels of agreement between the self-report and interviewer-rated retrospective reports of childhood trauma have been observed in individuals with first-episode psychosis and population-based controls (Gayer-Anderson *et al*., 2020). Other types of childhood adversity not assessed (e.g. bullying victimisation) might also be relevant (Cunningham *et al*., 2016). Second, ESM is a burdensome research method, which may lead to sampling and selection bias. For example, one way this may have operated on findings may be that individuals with more intense symptoms may have been underrepresented in the sample, as assessment burden may have discouraged eligible individuals with severe symptoms from participation. In addition, it may be more challenging for individuals with more severe symptoms to reach sufficient compliance, which may lead to underrepresentation due to the exclusion of these participants. However, we found no differences in clinical characteristics at baseline when comparing participants included in the analysis to individuals for whom ESM data were not available. Third, follow-up intervals varied, which was accounted for by controlling for time to follow-up and conducting sensitivity analyses with a restricted sample (leading to similar results in terms of magnitude of associations but some variation in statistical significance due to varying sample sizes). Fourth, unmeasured confounders (e.g. polygenic risk) may have influenced the reported findings. Fifth, although an increasingly common finding in the field (Simon *et al*., 2011; Hartmann *et al*., 2016; Nelson *et al*., 2016; Formica *et al*., 2020), we need to consider the small number of nine individuals (11%) who transitioned to psychosis within the follow-up period. The findings should therefore be re-evaluated in a larger sample with higher transition rates. In addition, comorbidity, especially comorbid major depressive and anxiety disorders, should be taken into account. Therefore, all analyses were controlled for comorbid major depressive and anxiety disorders. Sixth, the use of a composite stress measure should be critically discussed. In line with previous studies, we aggregated event-related, activity-related and social stress for each beep to reduce multiple testing (Pries *et al*., 2020; Klippel *et al*., 2021). Still, type I error should be taken into account when interpreting the results.

### Comparison with previous research

In accordance with previous ESM studies, we found that momentary stress was associated with small to moderate increases in negative affect and psychotic experiences and moderate decreases in positive affect in UHR individuals (Reininghaus *et al*., 2016*b*; van der Steen *et al*., 2017).

When considering the role of childhood trauma and stress reactivity in clinical trajectories, several possibilities of how these may combine with each other may be relevant (Schwartz and Susser, 2006; Morgan *et al*., 2014). Following Morgan *et al*. (2014), we investigated both effect modification and mediation in the same analyses. In accordance with suggested models and recent ESM studies, we found that childhood trauma amplifies reactivity to minor stress in daily life (Hammen *et al*., 2000; Myin-Germeys *et al*., 2001; Kendler *et al*., 2004; Morgan *et al*., 2010; Reininghaus *et al*., 2016*a*; Rauschenberg *et al*., 2017). Furthermore, we found some evidence that stress reactivity predicted clinical outcomes at follow-up. This extends findings from a previous ESM study in the general population and an observational study in patients with first-episode psychosis (Conus *et al*., 2009; Collip *et al*., 2013). Going one step further, there was some evidence that stress reactivity mediated the association of childhood trauma and clinical outcomes at follow-up. High levels of childhood trauma were associated with an increased stress reactivity, which, in turn, was associated with worse clinical outcomes at follow-up. Hence, this tentatively suggests that childhood trauma may both modify stress reactivity and exert detrimental effects via stress reactivity and push individuals along more severe clinical trajectories. Overall, this adds evidence in support of a mediated synergy model (Hafeman and Schwartz, 2009).

## Conclusion

Taken together, our findings underscore the relevance of reactivity to daily stressors as a putative mechanism linking childhood trauma with clinical outcomes in UHR individuals. Adding evidence to the mediated synergy model, the study suggests early adversity in childhood links to more severe clinical trajectories via, and in interaction with, subsequently elevated stress reactivity in adulthood. Therefore, the findings underline the relevance of ecological momentary interventions targeting stress reactivity in daily life (e.g. EMIcompass, a transdiagnostic ecological momentary intervention for improving resilience in youth; Schick *et al*., 2020) as an important next step towards improving clinical outcomes in UHR individuals at an early stage (Addington *et al*., 2012; Myin-Germeys *et al*., 2016, 2018; Reininghaus, 2018; Reininghaus *et al*., 2019).

## Data Availability

The data will not be available due to their sensitive nature (UHR status) and the fact that participants did not provide consent to the publication.

## Footnotes

1 This counterintuitive finding can be explained by centre and time to follow-up acting as suppressor variables (i.e. these variables suppressed, in part the variance of the independent variable of psychotic stress reactivity). When we examined the associations among independent and outcome variables, we found the typical pattern as it would be expected for suppressor effects: centre and time to follow-up were not correlated with the outcome variable but showed substantial associations with other independent variables.

## Supplementary material

For supplementary material accompanying this paper visit http://dx.doi.org/10.1017/S2045796021000251. click here to view supplementary material

## Appendix

### EU-GEI High Risk Study – group author list

Philip McGuire^1^, Lucia R. Valmaggia^2^, Matthew J. Kempton^1^, Maria Calem^1^, Stefania Tognin^1^, Gemma Modinos^1^, Lieuwe de Haan^3^, Mark van der Gaag^4,5^, Eva Velthorst^3,6^, Tamar C. Kraan^3^, Nadine Burger^5^, Daniella S. van Dam^3^, Neus Barrantes-Vidal^7,8,9,10^, Tecelli Domínguez-Martínez^7^, Paula Cristóbal-Narváez^7^, Thomas R. Kwapil^8^, Manel Monsonet-Bardají^7^, Lídia Hinojosa^7^, Anita Riecher-Rössler^11^, Stefan Borgwardt^11^, Charlotte Rapp^11^, Sarah Ittig^11^, Erich Studerus^11^, Renata Smieskova^11^, Rodrigo Bressan^12^, Ary Gadelha^12^, Elisa Brietzke^13^, Graccielle Asevedo^12^, Elson Asevedo^12^, Andre Zugman^12^, Stephan Ruhrmann^14^, Dominika Gebhard^14^, Julia Arnhold^15^, Joachim Klosterkötter^14^, Dorte Nordholm^16^, Lasse Randers^16^, Kristine Krakauer^16^, Tanya Louise Naumann^16^, Louise Birkedal Glenthøj^16^, Merete Nordentoft^16^, Marc De Hert^17^, Ruud van Winkel^17^, Barnaby Nelson^18^, Patrick McGorry^18^, Paul Amminger^18^, Christos Pantelis^18^, Athena Politis^18^, Joanne Goodall^18^, Gabriele Sachs^19^, Iris Lasser^19^, Bernadette Winklbaur^19^, Mathilde Kazes^20^, Claire Daban^20^, Julie Bourgin^20^, Olivier Gay^20^, Célia Mam-Lam-Fook^20^, Marie-Odile Krebs^20^, Bart P. Rutten^21^, Jim van Os^1,22^

^1^Department of Psychosis Studies, Institute of Psychiatry, King's College London, London, UK; ^2^Department of Psychology, Institute of Psychiatry, Psychology & Neuroscience, King's College London, London, UK; ^3^Department of Psychiatry, Academic Medical Center, University of Amsterdam, Amsterdam, The Netherlands; ^4^Department of Clinical Psychology, VU University and Amsterdam; Public Mental Health Research Institute, Amsterdam, The Netherlands; ^5^Department of Psychosis Research, Parnassia Psychiatric Institute, The Hague, The Netherlands; ^6^Department of Psychiatry, Icahn School of Medicine at Mount Sinai, New York, USA; ^7^Departament de Psicologia, Clínica i de la Salut, Universitat Autònoma de Barcelona, Barcelona, Spain; ^8^Departament de Salut Mental, Sant Pere Claver-Fundació Sanitària, Barcelona, Spain; ^9^Spanish Mental Health Research Network, CIBERSAM, Madrid, Spain; ^10^Department of Psychology, University of North Carolina at Greensboro, Greensboro, USA; ^11^Center for Gender Research and Early Detection, Psychiatric University Clinics Basel, Basel, Switzerland; ^12^LiNC – Lab Interdisciplinar Neurociências Clínicas, Depto Psiquiatria, Escola Paulista de Medicina, Universidade Federal de São Paulo – UNIFESP, São Paulo, Brazil; ^13^Pogram for cognition and Intervention in Individuals in At-Risk Mental States (PRISMA), Department of Psychiatry, Universidade Federal de São Paulo, São Paulo, Brazil; ^14^Department of Psychiatry and Psychotherapy, University of Cologne, Cologne, Germany; ^15^Psyberlin, Berlin, Germany; ^16^Mental Health Center Copenhagen and Center for Clinical Intervention and Neuropsychiatric Schizophrenia Research, CINS, Mental Health Center Glostrup, Mental Health Services in the Capital Region of Copenhagen, University of Copenhagen, Copenhagen, Denmark; ^17^Department of Neuroscience, University Psychiatric Centre, Catholic University Leuven, Leuven, Belgium; ^18^Centre for Youth Mental Health, University of Melbourne, Melbourne, Australia; ^19^Department of Psychiatry and Psychotherapy, Medical University of Vienna, Vienna, Austria; ^20^University Paris Descartes, Hôpital Sainte-Anne, C'JAAD, Service Hospitalo-Universitaire, Inserm U894, Institut de Psychiatrie, Paris, France; ^21^Department of Psychiatry and Neuropsychology, School for Mental Health and Neuroscience, Maastricht University Medical Centre, Maastricht, The Netherlands and ^22^Department of Psychiatry and Psychology, Maastricht University Medical Center, Maastricht, The Netherlands.
