# Supplementary material for: Stress reactivity as a putative mechanism linking childhood trauma with clinical outcomes in individuals at ultra-high-risk for psychosis: Findings from the EU-GEI High Risk Study
Source: Epidemiol Psychiatr Sci. 2021 May 28;30:e40. doi: 10.1017/S2045796021000251 (PMC8193966; doi:10.1017/S2045796021000251)
Supplement: Supplementary file 1 [file S2045796021000251sup001.docx]

**SUPPLEMENTARY MATERIALS**

[**1.** **Graphic illustration of the hypotheses tested in the current study** 3](#_Toc67056631)

[**2.** **Overview of the measures used in the current study** 4](#_Toc67056632)

[**3.** **Data quality of clinical outcome measures** 5](#_Toc67056633)

[**4.** **Restricted sample – unadjusted analyses** 6](#_Toc67056634)

[**4.1 Method** 6](#_Toc67056635)

[**4.2 Results** 6](#_Toc67056636)

[*4.2.1 Basic sample and clinical characteristics* 6](#_Toc67056637)

[*4.2.2 Stress reactivity and clinical outcomes at follow-up (H3)* 8](#_Toc67056638)

[*4.2.3 Emotional and psychotic stress reactivity as mediators of the association of childhood trauma and clinical outcomes (H4)* 10](#_Toc67056639)

[**5.** **Restricted sample – adjusted analyses** 12](#_Toc67056640)

[**5.1 Method** 12](#_Toc67056641)

[**5.2 Results** 12](#_Toc67056642)

[*5.2.1 Stress reactivity and clinical outcomes at follow-up (H3)* 12](#_Toc67056643)

[*5.2.2 Emotional and psychotic stress reactivity as mediators of the association of childhood trauma and clinical outcomes (H4)* 14](#_Toc67056644)

[**6.** **Unadjusted analysis in the full sample** 16](#_Toc67056645)

[**6.1 Method** 16](#_Toc67056646)

[**6.2 Results** 16](#_Toc67056647)

[*6.2.1 Association between momentary stress, affect and psychotic experiences (H1)* 16](#_Toc67056648)

[*6.2.2 Association between momentary stress, affect and psychotic experiences by childhood trauma (H2)* 16](#_Toc67056649)

[*6.2.3 Stress reactivity and clinical outcomes at follow-up (H3)* 17](#_Toc67056650)

[*6.2.4 Emotional and psychotic stress reactivity as mediators of the association of childhood trauma and clinical outcomes (H4)* 19](#_Toc67056651)

[**7.** **Exploratory analyses: The role of transition** 21](#_Toc67056652)

[**7.1 Does transition moderate the effect of momentary stress on affect and psychotic experiences?** 21](#_Toc67056653)

[*7.1.1 Method* 21](#_Toc67056654)

[*7.1.2 Results* 21](#_Toc67056655)

[**7.2 Do emotional and psychotic stress reactivity mediate the association of childhood trauma and transition?** 23](#_Toc67056656)

[*7.2.1 Method* 23](#_Toc67056657)

[*7.2.2 Results* 24](#_Toc67056658)

[**8.** **Examining the structural validity of the ESM items** 25](#_Toc67056659)

[**8.1 Method** 25](#_Toc67056660)

[**8.2 Results** 25](#_Toc67056661)

# **Graphic illustration of the hypotheses tested in the current study**

Hypothesis 1:


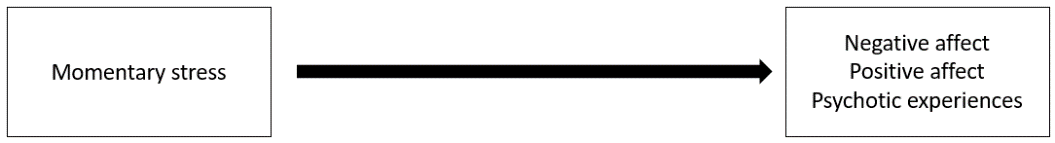


Hypothesis 2:


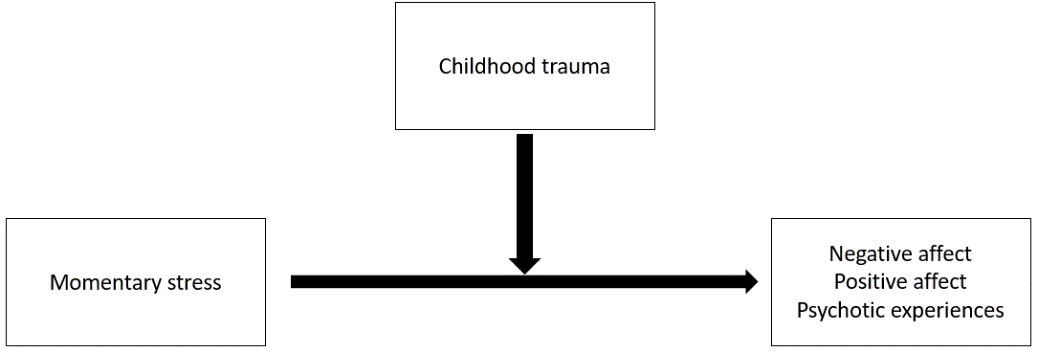


Hypothesis 3:


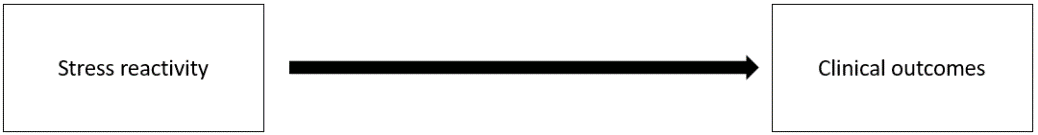


Hypothesis 4:


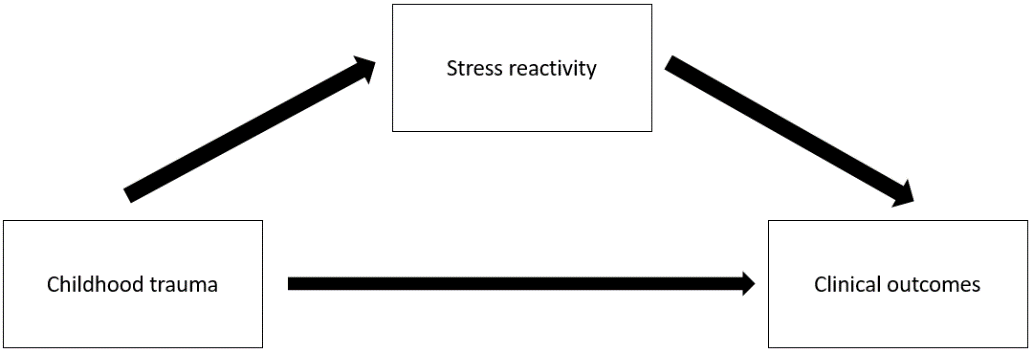


Figure 1S. Graphic of hypotheses tested.

# **Overview of the measures used in the current study**

**Table S1.** Data collection

| **Experience sampling ^a^** | |
| --- | --- |
| Momentary stress | Momentary stress was defined as minor disturbances occurring throughout the day based on previous ESM studies (Myin-Germeys *et al.*, 2001; Palmier-Claus *et al.*, 2012). We used a composite stress measure (row mean) consisting of items assessing event-related, activity-related and social stress to avoid multiple testing (Pries *et al.*, 2020; Klippel *et al.*, submitted). *Event-related stress:* Participants were asked to rate the most important event since the last beep on a 7-point Likert scale ranging from -3 (very unpleasant) to 3 (very pleasant). We recoded this item so that higher ratings indicate higher levels of stress (-3 recoded as 7 and 3 recoded as 1). *Activity-related stress:* Participants were asked to indicate what they were doing just before the beep and answer three follow-up questions (‘I would prefer doing something else’, ‘This activity is difficult for me’, ‘I can do this well’ [reversed]) with a 7-point Likert scale ranging from 1 (not at all) to 7 (very much). *Social stress:* Participants were asked to specify categorically with whom they were spending time and then rated the following items on a 7-point Likert scale ranging from 1 (not at all) to 7 (very much): ‘I would prefer to be alone [if in company]/I would prefer to have company [if alone, reversed]’ and ‘I find being with these people pleasant [if in company]/ I find it pleasant to be alone [if alone]’. Previous research demonstrated good feasibility and reliability for the ESM items in UHR individuals and good concurrent validity with other stress measures (Myin-Germeys *et al.*, 2003; Palmier-Claus *et al.*, 2012). |
| Positive affect | Positive affect was measured by asking participants to rate how cheerful, relaxed, satisfied and enthusiastic they felt, rated on a Likert scale ranging from 1 (not at all) to 7 (very much). We found satisfying internal consistency (Cronbach’s α=.73). Mean scores were computed as overall measure of positive affect. |
| Negative affect | Negative affect was measured by asking participants to rate the extent to which they felt insecure, down, lonely, anxious and irritated on a Likert scale ranging from 1 (not at all) to 7 (very much). We found satisfying internal consistency (Cronbach’s α=.73). Mean scores were computed as overall measure of negative affect. |
| Psychotic experiences | The ESM psychosis measure was used to assess intensity of psychotic experiences. It consisted of 7 items (e.g. ‘I feel paranoid’, ‘I hear things that aren’t really there’) rated on a 7-point Likert scale ranging from 1 (not at all) to 7 (very much). Previous studies reported high levels of internal consistency and good concurrent validity with interviewer-rated measures of psychotic experiences (Reininghaus *et al.*, 2016; Myin-Germeys *et al.*, 2005). In the current study, the ESM items for psychotic experiences showed satisfying internal consistency (Cronbach’s α=.72). |
| **Childhood trauma** | |
| CTQ | The CTQ (Bernstein and Fink, 1998) assesses five types of childhood maltreatment (emotional, physical and sexual abuse, emotional and physical neglect) on a 5-point Likert scale (1=never true, 5=very often true). As the utility of a CTQ total score for clinical research and practice has been demonstrated, we calculated the sum of answers to all 25 questions (potential range 25-125) as a general measure of childhood trauma (Scher *et al.*, 2001). Good psychometric properties have been reported (Bernstein *et al.*, 1997; Bernstein *et al.*, 2003; Wingenfeld *et al.*, 2010; Scher *et al.*, 2001). In the current study, the CTQ total score showed excellent internal consistency (Cronbach’s α=.92). |
| **Clinical outcome measures** | |
| CGI | The CGI illness severity subscale is an expert rating of average illness severity during the last week ranging from 1 (normal, not at all ill) to 7 (among the most extremely ill patients; (Guy, 1976). |
| GAF | The GAF obtains ratings of burdening symptoms and disabilities in the last month on a scale from 100 (no symptoms/ superior functioning in a wide range of activities) to 1 (persistent danger of severely hurting self or others or serious suicidal act with clear expectation of death/ persistent inability to maintain minimal personal hygiene(American Psychiatric Association, 2002). |
| CAARMS | Symptoms were assessed on a scale from 0 (never, absent) to 7 (psychotic and severe) using the unusual thought content, perceptual abnormalities, anxiety and tolerance to normal stress subscales of the CAARMS (Yung *et al.*, 2005). |

^a^ ESM procedure: During an initial briefing, participants were asked to stop their activity and answer the questions each time the device emitted the beep signal. The ESM questionnaire was available to participants for the duration of 10 min after emission of the beep signal. Participants were contacted at least once during the assessment period to assess their adherence to instructions, identify any potential distress associated with the method, and maximize the number of observations per participant. At the end of the assessment period, participants’ reactivity to, and compliance with, the method was examined in a debriefing session. Participants were required to provide valid responses to at least one-third (i.e. 20 valid answers) of the emitted beeps to be included in the analysis (Delespaul *et al.*, 2002).

# **Data quality of clinical outcome measures**

To ensure data quality, extensive training on instruments and interview skills was provided. Initial assessments were reviewed, and possible difficulties were anticipated. In addition to the EU-GEI web-based training designed to control and increase inter-rater reliability, regular meetings were held to discuss case vignettes. Site visits were held in order to evaluate and standardize interviews. In addition, extensive, repetitious training procedures and reliability checks were conducted. Training videos of the most advanced instruments were updated regularly. For each of the training videos, a ‘golden standard score’ was determined through independent rating of the training videos by independent experienced researchers. In case of disagreement, the head of the training work package was consulted. Per instrument, we subsequently determined the maximum amount of errors/ deviation from the gold standard score the researcher was allowed, in order ‘pass’ the video.

# **Restricted sample – unadjusted analyses**

## **4.1 Method**

In Supplementary Material 4, we present unadjusted analyses in the restricted sample. The restricted sample only comprises participants, who returned within a +/- 6 month time interval around the expected follow-up time points. The analyses were conducted with varying sample sizes for illness severity, level of functioning and symptom burden.

## **4.2 Results**

### *4.2.1 Basic sample and clinical characteristics*

Table S2 gives an overview of relevant basic sample and clinical characteristics of the restricted sample at 1- (*N*=46) and 2-year follow-up (*N*=31).

**Table S2.** Basic sample and clinical characteristics for the restricted sample

|  | 1-year follow-up | 2-year follow-up |
| --- | --- | --- |
| Sample Size *N* | 46 | 31 |
| Age at baseline (years), mean (SD) | 23.9 (5.51) | 24.06 (5.25) |
| Gender *N*(%) |  |  |
| male | 21 (46%) | 12 (39%) |
| female | 25 (54%) | 19 61%) |
| Ethnicity *N*(%) |  |  |
| white | 31 (67%) | 23 (74%) |
| black | 9 (20%) | 4 (13%) |
| other | 6 (13%) | 4 (13%) |
| Comorbidity at baseline *N*(%) |  |  |
| Major depressive disorder *N*(%) | 14 (31%) | 11 (37%) |
| Current depressive episode *N*(%) | 11 (24%) | 8 (26%) |
| Bipolar disorder *N*(%) | 4 (9%) | 4 (13%) |
| Any anxiety disorder *N*(%) | 26 (57%) | 16 (52%) |
| Panic disorder *N*(%) | 12 (27%) | 6 (19%) |
| Panic disorder + agoraphobia *N*(%) | 4 (9%) | 1 (4%) |
| Agoraphobia only *N*(%) | 0 | 0 |
| Social phobia *N*(%) | 14 (30%) | 8 (26%) |
| Specific phobia *N*(%) | 9 (20%) | 5 (17%) |
| Generalized anxiety disorder *N*(%) | 7 (15%) | 5 (16%) |
| Not otherwise specified anxiety disorder *N*(%) | 1 (2%) | 0 |
| Obsessive-compulsive disorder *N*(%) | 2 (4%) | 3 (10%) |
| Posttraumatic stress disorder *N*(%) | 4 (9%) | 0 |
| Any eating disorder *N*(%) | 7 (15%) | 6 (19%) |
| Anorexia nervosa *N*(%) | 3 (7%) | 3 (10%) |
| Bulimia nervosa *N*(%) | 3 (7%) | 2 (6%) |
| Binge eating disorder *N*(%) | 1 (2%) | 1 (3%) |
| Any somatoform disorder *N*(%) | 1 (2%) | 1 (3%) |
| Somatization disorder *N*(%) | 0 | 0 |
| Chronic pain *N*(%) | 0 | 0 |
| Hypochondriasis *N*(%) | 1 (2%) | 1 (3%) |
| Body dismorph disorder *N*(%) | 0 | 1 |
| Childhood trauma questionnaire total score at baseline, mean (SD) | 49.70 (16.63) | 47.74 (13.41) |
| Clinical global impression  illness severity, mean (SD) | 3.73 (1.16) | 3.87 (1.22) |
| Global assessment of functioning |  |  |
| disability, mean (SD) | 56.15 (12.65) | 57.00 (12.09) |
| Comprehensive Assessment of At Risk Mental States |  |  |
| Unusual thought content, mean (SD) | 2.91 (1.94) | 2.58 (1.77) |
| Perceptual abnormalities, mean (SD) | 3.13 (1.75) | 3.03 (1.54) |
| Anxiety, mean (SD) | 3.43 (1.00) | 3.45 (0.62) |
| Tolerance to normal stress, mean (SD) | 2.22 (1.87) | 2.29 (1.66) |

*Note.* ESM = experience sampling method, *N* = sample size, SD = standard deviation. Comorbidity: Participants were diagnosed with a comorbid disorder, if classification criteria were fulfilled. Thus, one participant can be diagnosed with multiple comorbid disorders. Sample sizes based on valid restricted GAF at follow-up.

### *4.2.2 Stress reactivity and clinical outcomes at follow-up (H3)*

As displayed in Table S3, in the restricted sample, illness severity at follow-up was not predicted by emotional or psychotic stress reactivity. However, decreased positive affect in response to stress predicted level of functioning at 1-year follow-up (*B*=7.16, 95% CI 1.22 – 13.10, *P*=.019). Increased negative affect (*B*=1.45, 95% CI 0.75 – 2.14, *P*<.001) and increased psychotic experiences in response to stress (*B*=1.11, 95% CI 0.35– 1.88, *P*=.006) predicted perceptual abnormalities at 1-year follow-up. In addition, decreased positive affect in response to stress predicted anxiety at 1-year follow-up (*B*=-0.83, 95% CI -1.59– -0.07, *P*=.032).

|  | **Clinical Outcomes** | | | | | | | |
| --- | --- | --- | --- | --- | --- | --- | --- | --- |
|  | **Illness severity (CGI)** | | | | **Level of functioning Disability (GAF)** | | | |
|  | 1-year follow-up (*N*=42) | | 2-year follow-up (*N*=32) | | 1-year follow-up (*N*=46) | | 2-year follow-up (*N*=31) | |
|  | *B* (95% CI) | *P* | *B* (95% CI) | *P* | *B* (95% CI) | *P* | *B* (95% CI) | *P* |
| **Predictor: Emotional reactivity (increased negative affect in response to stress)** | | | | | | | | |
| Outcome at baseline | 0.74  (0.50 – 0.97) | <.001 | 0.45  (0.08 – 0.82) | .020 | 0.40  (0.12 – 0.69) | .007 | 0.49  (0.11 – 0.87) | .013 |
| Emotional reactivity | 0.28  (-0.19 – 0.74) | .234 | -0.08  (-0.71 – 0.54) | .793 | -5.30  (-10.97 – 0.37) | .066 | 1.24  (-5.32 – 7.79) | .701 |
| **Predictor: Emotional reactivity (decreased positive affect in response to stress)** | | | | | | | | |
| Outcome at baseline | 0.74  (0.49 – 0.99) | <.001 | 0.38  (0.01 – 0.74) | .045 | 0.36  (0.08 – 0.63) | .013 | 0.50  (0.12 – 0.87) | .011 |
| Emotional reactivity | -0.21  (-0.74 – 0.31) | .418 | -0.48  (-1.18 – 0.21) | .162 | 7.16  (1.22 – 13.10) | .019 | 2.01  (-5.15 – 9.16) | .571 |
| **Predictor: Psychotic reactivity (increased psychotic experiences in response to stress)** | | | | | | | | |
| Outcome at baseline | 0.78  (0.54 – 1.01) | <.001 | 0.44  0.08 – 0.81) | .019 | 0.42  (0.12 – 0.71) | .007 | 0.48  (0.09 – 0.87) | .018 |
| Psychotic reactivity | 0.01  (-0.44 – 0.45) | .980 | -0.16  (-0.77 – 0.45) | .588 | -3.28  (-9.00 – 2.43) | .253 | 1.62  (-5.29 – 8.53) | .635 |
|  | **Unusual thought content (CAARMS)** | | | | **Perceptual abnormalities (CAARMS)** | | | |
|  | 1-year follow-up (*N*=40) | | 2-year follow-up (*N*=30) | | 1-year follow-up (*N*=40) | | 2-year follow-up (*N*=30) | |
|  | *B* (95% CI) | *P* | *B* (95% CI) | *P* | *B* (95% CI) | *P* | *B* (95% CI) | *P* |
| **Predictor: Emotional reactivity (increased negative affect in response to stress)** | | | | | | | | |
| Outcome at baseline | 0.46  (0.16 – 0.76) | .004 | 0.25  (-0.17 – 0.67) | .231 | 0.42  (0.18 – 0.67) | .001 | 0.43  (0.00 – 0.87) | .052 |
| Emotional reactivity | 0.66  (-0.27 – 1.59) | .160 | 0.45  (-0.81 – 1.70) | .474 | 1.45  (0.75 – 2.14) | <.001 | -0.20  (-1.32 – 0.92) | .720 |
| **Predictor: Emotional reactivity (decreased positive affect in response to stress)** | | | | | | | | |
| Outcome at baseline | 0.49  (0.19 – 0.79) | .002 | 0.30  (-0.11 – 0.70) | .146 | 0.48  (0.20 – 0.77) | .002 | 0.47  (0.03 – 0.91) | .039 |
| Emotional reactivity | -0.70  (-1.69 – 0.29) | .161 | -0.18  (-1.44 – 1.09) | .778 | -0.68  (-1.55 – 0.19) | .124 | -0.45  (-1.63 – 0.73) | .438 |
| **Predictor: Psychotic reactivity (increased psychotic experiences in response to stress)** | | | | | | | | |
| Outcome at baseline | 0.43  (0.12 – 0.74) | .008 | 0.20  (-0.23 – 0.64) | .351 | 0.36  (0.09 – 0.63) | .011 | 0.41  (-0.03 – 0.84) | .066 |
| Psychotic reactivity | 0.60  (-0.34 – 1.54) | .206 | 0.70  (-0.62 – 2.02) | .285 | 1.11  (0.35 – 1.88) | .006 | 0.39  (-0.74 – 1.52) | .488 |
|  | **Anxiety (CAARMS)** | | | | **Tolerance to normal stress (CAARMS)** | | | |
|  | 1-year follow-up (*N*=40) | | 2-year follow-up (*N*=30) | | 1-year follow-up (*N*=40) | | 2-year follow-up (*N*=30) | |
|  | *B* (95% CI) | *P* | *B* (95% CI) | *P* | *B* (95% CI) | *P* | *B* (95% CI) | *P* |
| **Predictor: Emotional reactivity (increased negative affect in response to stress)** | | | | | | | | |
| Outcome at baseline | 0.32  (-0.09 – 0.73) | .122 | 0.90  (0.08 – 1.73) | .033 | 0.31  (0.05 – 0.56) | 0.18 | 0.18  (-0.20 – 0.55) | .343 |
| Emotional reactivity | 0.51  (-0.27 – 1.28) | .194 | -0.72  (1.,87 – 0.44) | .216 | -0.08  (-0.84 – 0.68) | .833 | -0.04  (-1.51 – 1.07) | .942 |
| **Predictor: Emotional reactivity (decreased positive affect in response to stress)** | | | | | | | | |
| Outcome at baseline | 0.33  (-0.05 – 0.71) | .084 | 0.70  (-0.08 – 1.48) | .077 | 0.30  (0.05 – 0.55) | .019 | 0.14  (-0.21 – 0.49) | .412 |
| Emotional reactivity | -0.83  (-1.59 – -0.07) | .032 | -0.08  (-1.22 – 1.07) | .893 | 0.22  (-0.58 – 1.02) | .580 | -0.54  (-1.62 – 0.54) | .316 |
| **Predictor: Psychotic reactivity (increased psychotic experiences in response to stress)** | | | | | | | | |
| Outcome at baseline | 0.38  (-0.01 – 0.77) | .058 | 0.76  (0.02 – 1.51) | .045 | 0.31  (0.05 – 0.56) | .019 | 0.22  (-0.13 – 0.58) | .209 |
| Psychotic reactivity | 0.43  (-0.29 – 1.16) | .234 | -0.86  (-1.93 – 0.20) | .106 | -0.06  (-0.38 – 1.00) | .365 | -0.54  (-1.62 – 0.54) | .312 |

**Table S3.** Restricted sample: Clinical Outcomes at 1- and 2-year follow-up predicted by emotional and psychotic stress reactivity at baseline and clinical outcome at baseline
*Note*. Illness severity assessed with the Clinical Global Impression Scale (CGI). Level of functioning assessed with the Global Assessment of Functioning Scale (GAF). *N* = sample size, 95% CI = 95% confidence interval

### *4.2.3 Emotional and psychotic stress reactivity as mediators of the association of childhood trauma and clinical outcomes (H4)*

Table S4 shows the unadjusted results on emotional and psychotic stress reactivity as mediators of the association of childhood trauma and clinical outcomes in the restricted sample. It displays total, direct and indirect effects of childhood trauma, emotional and psychotic stress reactivity on illness severity, level of functioning and CAARMS symptoms at follow-up. The association of illness severity at 1-year follow-up and childhood trauma was mediated by decreased positive affect in response to stress (indirect effect: *B*=0.16, 95% CI 0.00 – 0.32, *P*=.049). The association of childhood trauma and unusual thought content at 1-year follow-up was mediated by psychotic reactivity (indirect effect: *B*=0.34, 95% CI 0.01 – 0.67, *P*=.046). Furthermore, the association of childhood trauma and perceptual abnormalities at 1-year follow-up was mediated by increase negative affect (indirect effect: *B*=0.46, 95% CI 0.14 – 0.78, *P*=.005) and increase psychotic experiences in response to stress (indirect effect: *B*=0.47, 95% CI 0.15 – 0.79, *P*=.004).

|  | **Clinical Outcomes** | | | | | | | |
| --- | --- | --- | --- | --- | --- | --- | --- | --- |
|  | **Illness severity (CGI)** | | | | **Level of functioning Disability (GAF)** | | | |
|  | 1-year follow-up (*N*=43) | | 2-year follow-up (*N*=33) | | 1-year follow-up (*N*=46) | | 2-year follow-up (*N*=31) | |
|  | *B* (95% CI) | *P* | *B* (95% CI) | *P* | *B* (95% CI) | *P* | *B* (95% CI) | *P* |
| **Mediator: Emotional reactivity (increased negative affect in response to stress)** | | | | | | | | |
| Total effect | 0.43 (0.05 – 0.81) | .027 | -0.11 (-0.59 – 0.37) | .645 | -3.40 (-7.13 – 0.33) | .074 | 1.36 (-4.06 – 6.78) | .623 |
| Direct effect | 0.24 (-0.12 – 0.60) | .191 | -0.15 (-0.66 – 0.36) | .570 | -2.34 (-6.09 – 1.41) | .222 | 0.85 (-4.93 – 6.63) | .773 |
| Indirect effect | 0.19 (-0.01 – 0.38) | .059 | 0.03 (-0.15 – 0.22) | .709 | -1.06 (-2.81 – 0.69) | .235 | 0.51 (-1.50 – 2.52) | .618 |
| **Mediator: Emotional reactivity (decreased positive affect in response to stress)** | | | | | | | | |
| Total effect | 0.40 (0.03 – 0.77) | .032 | -0.12 (-0.57 – 0.34) | .619 | -3.49 (-7.12 – 0.15) | .060 | 1.50 (-3.90 – 6.91) | .586 |
| Direct effect | 0.24 (-0.11 – 0.59) | .178 | -0.27 (-0.73 – 0.19) | .254 | -2.13 (-5.69 – 1.43) | .241 | 2.13 (-3.54 – 7.79) | .462 |
| Indirect effect | 0.16 (0.00 – 0.32) | .049 | 0.15 (-0.01 – 0.31) | .064 | -1.36 (-2.82 – 0.11) | .069 | -0.62 (-2.15 – 0.90) | .422 |
| **Mediator: Psychotic reactivity (increased psychotic experiences in response to stress)** | | | | | | | | |
| Total effect | 0.37 (-0.02 – 0.75) | .061 | -0.12 (-0.60 – 0.36) | .624 | -3.00 (-6.74 – 0.74) | .116 | 1.89 (-3.58 – 7.35) | .499 |
| Direct effect | 0.28 (-0.10 – 0.66) | .145 | -0.10 (-0.60 – 0.40) | .692 | .2,57 (-6.75 – 1.01) | .147 | 0.77 (-4.70 - .624) | .782 |
| Indirect effect | 0.08 (-0.11 – 0.27) | .394 | -0.02 (-0.22 – 0.18) | .840 | -0.13 (-1.98 – 1.71) | .888 | 1.11 (-1.08 – 3.31) | .320 |
|  | **Unusual thought content (CAARMS)** | | | | **Perceptual abnormalities (CAARMS)** | | | |
|  | 1-year follow-up (*N*=40) | | 2-year follow-up (*N*=31) | | 1-year follow-up (*N*=40) | | 2-year follow-up (*N*=30) | |
|  | *B* (95% CI) | *P* | *B* (95% CI) | *P* | *B* (95% CI) | *P* | *B* (95% CI) | *P* |
| **Mediator: Emotional reactivity (increased negative affect in response to stress)** | | | | | | | | |
| Total effect | -0.12 (-0.72 – 0.48) | .697 | 0.54 (-0.37 – 1.45) | .242 | 0.16 (-0.35 – 0.67) | .549 | -0.14 (-1.02 – 0.74) | .755 |
| Direct effect | -0.37 (-0.96 – 0.22) | .220 | -0.37 (-0.52 – 1.25) | .416 | -0.30 (-0.75 – 0.15) | .186 | -0.09 (-0.95 – 0.77) | .838 |
| Indirect effect | 0.24 (-0.06 – 0.55) | .110 | 0.18 (-0.15 – 0.50) | .287 | 0.46 (0.14 – 0.78) | .005 | -0.05 (-0.36 – 0.26) | .746 |
| **Mediator: Emotional reactivity (decreased positive affect in response to stress)** | | | | | | | | |
| Total effect | -0.19 (-0.79 – 0.41) | .532 | 0.43 (-0.47 – 1.34) | .348 | -0.03 (-0.57 – 0.50) | .899 | -0.08 (-0.95 – 0.78) | .850 |
| Direct effect | -0.31 (-0.91 – 0.28) | .299 | 0.40 (-0.50 – 1.30) | .379 | -0.14 (-0.67 – 0.39) | .612 | -0.12 (-0.98 – 0.74) | .785 |
| Indirect effect | 0.12 (-0.09 – 0.34) | .265 | 0.03 (-0.20 – 0.26) | .801 | 0.10 (-0.09 – 0.30) | .296 | 0.04 (-0.18 – 0.26) | .746 |
| **Mediator: Psychotic reactivity (increased psychotic experiences in response to stress)** | | | | | | | | |
| Total effect | -0.09 (-0.69 – 0.50) | .762 | 0.68 (-0.24 – 1.60) | .147 | 0.13 (-0.38 – 0.64) | .610 | 0.05 (-0.84 – 0.95) | .906 |
| Direct effect | -0.43 (-1.01 – 0.15) | .147 | 0.39 (-0.47 – 1.25) | .373 | -0.33 (-0.81 – 0.14) | .164 | -0.12 (-0.96 – 0.72) | .784 |
| Indirect effect | 0.34 (0.01 – 0.67) | .046 | 0.29 (-0.07 – 0.66) | .118 | 0.47 (0.15 – 0.79) | .004 | 0.17 (-0.17 – 0.52) | .327 |
|  | **Anxiety (CAARMS)** | | | | **Tolerance to normal stress (CAARMS)** | | | |
|  | 1-year follow-up (*N*=40) | | 2-year follow-up (*N=31*) | | 1-year follow-up (*N*=40) | | 2-year follow-up (*N*=31) | |
|  | *B* (95% CI) | *P* | *B* (95% CI) | *P* | *B* (95% CI) | *P* | *B* (95% CI) | *P* |
| **Mediator: Emotional reactivity (increased negative affect in response to stress)** | | | | | | | | |
| Total effect | 0.25 (-0.19 – 0.70) | .268 | -0.24 (-1.10 – 0.62) | .580 | -0.07 (-0.53 – 0.40) | .778 | 0.12 (-0.66 – 0.91) | .756 |
| Direct effect | .006 (-0.37 – 0.49) | .792 | -0.19 (-1.02 – 0.65) | .657 | -0.08 (-0.55 – 0.38) | .727 | 0.09 (-0.68 – 0.85) | .826 |
| Indirect effect | 0.19 (-0.03 – 0.42) | .093 | -0.05 (-0.35 – 0.24) | .725 | 0.02 (-0.20 – 0.23) | .888 | 0.04 (-0.23 – 0.31) | .778 |
| **Mediator: Emotional reactivity (decreased positive affect in response to stress)** | | | | | | | | |
| Total effect | 0.24 (-0.19 – 0.66) | .283 | -0.19 (-1.03 – 0.65) | .660 | -0.10 (-0.55 – 0.36) | .679 | 0.17 (-.09 – 0.92) | .668 |
| Direct effect | 0.05 (-0.36 – 0.47) | .796 | -0.21 (-1.05 – 0.62) | .616 | -0.05 (-0.51 – 0.41) | .828 | 0.05 (-0.70 – 0.80) | .899 |
| Indirect effect | 0.18 (0.00 – 0.37) | .055 | 0.03 (-0.19 – 0.24) | .817 | -0.05 (-0.20 – 0.11) | .572 | 0.12 (-0.09 – 0.32) | .263 |
| **Mediator: Psychotic reactivity (increased psychotic experiences in response to stress)** | | | | | | | | |
| Total effect | 0.22 (-0.22 – 0.67) | .329 | -0.41 (-1.28 – 0.45) | .347 | -0.06 (-0.53 – 0.40) | .790 | 0.00 (-0.81 – 0.80) | .996 |
| Direct effect | 0.07 (-0.38 – 0.51) | .767 | -0.18 (-0.99 – 0.62) | .656 | -0.09 (-0.56 – 0.38) | .710 | 0.11 (-0.65 – 0.86) | .784 |
| Indirect effect | 0.16 (-0.08 – 0.39) | .200 | -0.23 (-0.57 – 0.11) | .179 | 0.03 (-0.21 – 0.27) | .831 | -0.11 (-0.41 – 0.20) | .488 |

**Table S4**. Restricted sample: Emotional and psychotic stress reactivity as mediators of the association of childhood trauma and clinical outcomes

*Note*. Childhood trauma assessed with the CTQ. Illness severity assessed with the Clinical Global Impression Scale (CGI). Level of functioning assessed with the Global Assessment of Functioning Scale (GAF). Unusual thought content, perceptual abnormalities, anxiety and tolerance to normal stress assessed with the Comprehensive Assessment of At Risk Mental State (CAARMS). *N* = sample size, 95% CI = 95% confidence interval.

# **Restricted sample – adjusted analyses**

## **5.1 Method**

In Supplementary Material 5, we present adjusted analyses in the restricted sample. The restricted sample only comprises participants, who returned within a +/- 6 month time interval around the expected follow-up time points. The analyses were conducted with varying sample sizes for illness severity, level of functioning and symptom burden. The analyses are adjusted for age, gender, ethnicity, centre, comorbid major depressive and anxiety disorders and time to follow-up.

## **5.2 Results**

### *5.2.1 Stress reactivity and clinical outcomes at follow-up (H3)*

As displayed in Table S5, in the restricted sample, illness severity at follow-up was not predicted by emotional or psychotic stress reactivity. However, decreased positive affect in response to stress predicted level of functioning at 1-year follow-up (*B*=6.64, 95% CI 0.14– 13.13, *P*=.046). Increased negative affect in response to stress predicted unusual thought content at 2-year follow-up (*B*=1.83, 95% CI 0.17– 3.48, *P*=.033). In addition, perceptual abnormalities at 1-year follow-up were predicted by emotional (negative affect: *B=*1.31, 95% CI 0.49– 2.13, *P*=.003; positive affect: *B*=-1.09, 95% CI -1.96– -0.23, *P*=.015) and psychotic stress reactivity (*B*=1.09, 95% CI 0.18– 2.00, *P*=.020). More intense emotional and psychotic reactivity was associated with higher symptom burden and lower level of functioning.

**Table S5.** Restricted sample: Clinical Outcomes at 1- and 2-year follow-up predicted by emotional and psychotic stress reactivity at baseline and clinical outcome at baseline

|  | **Clinical Outcomes** | | | | | | | |
| --- | --- | --- | --- | --- | --- | --- | --- | --- |
|  | **Illness severity (CGI)** | | | | **Level of functioning Disability (GAF)** | | | |
|  | 1-year follow-up (*N*=41) | | 2-year follow-up (*N*=31) | | 1-year follow-up (*N*=45) | | 2-year follow-up (*N*=) | |
|  | *B* (95% CI) | *P* | *B* (95% CI) | *P* | *B* (95% CI) | *P* | *B* (95% CI) | *P* |
| **Predictor: Emotional reactivity (increased negative affect in response to stress)** | | | | | | | | |
| Outcome at baseline | 0.71  (0.46 – 0.96) | <.001 | 0.46  (-0.07 – 0.98) | .085 | 0.42  (0.07 – 0.76) | .019 | 0.56  (0.03 – 1.08) | .040 |
| Emotional reactivity | 0.36  (-0.08 – 0.80) | .105 | -0.34  (-1.28 – 1.83) | .338 | -3.89  (-10.86 -3.08) | .265 | 5.60  (-4.36 – 15.57) | .255 |
| **Predictor: Emotional reactivity (decreased positive affect in response to stress)** | | | | | | | | |
| Outcome at baseline | 0.73  (0.49 – 0.97) | <.001 | 0.29  (-0.26 – 0.83) | .284 | 0.41  (0.09 – 0.74) | .015 | 0.48  (-0.05 – 1.02) | .075 |
| Emotional reactivity | -0.42  (-0.86 – 0.02) | .061 | -0.34  (-1.30 – 0.62) | .474 | 6.64  (0.14 – 13.13) | .046 | -1.22  (-10.45 – 7.99) | .784 |
| **Predictor: Psychotic reactivity (increased psychotic experiences in response to stress)** | | | | | | | | |
| Outcome at baseline | 0.75  (0.47 – 1.03) | <.001 | 0.44  (-0.06 – 0.95) | .083 | 0.45  (0.11 – 0.79) | .012 | 0.51  (0.01 – 1.00) | .046 |
| Psychotic reactivity | 0.13  (-0.36 – 0.61) | .598 | -0.37  (-1.33 – 0.60) | .440 | -1.11  (-8.21 – 5.99) | .753 | 7.39  (-3.20 – 17.98) | .161 |
|  | **Unusual thought content (CAARMS)** | | | | **Perceptual abnormalities (CAARMS)** | | | |
|  | 1-year follow-up (*N*=39) | | 2-year follow-up (*N*=30) | | 1-year follow-up (*N*=39) | | 2-year follow-up (*N*=29) | |
|  | *B* (95% CI) | *P* | *B* (95% CI) | *P* | *B* (95% CI) | *P* | *B* (95% CI) | *P* |
| **Predictor: Emotional reactivity (increased negative affect in response to stress)** | | | | | | | | |
| Outcome at baseline | 0.53  (0.19 – 0.86) | .004 | -0.16  (-0.65 – 0.32) | .483 | 0.41  (0.14 – 0.67) | .004 | 0.54  (-0.04 – 1.12) | .066 |
| Emotional reactivity | 1.06  (0.03 – 2.09) | .044 | 1.83  (0.17 – 3.48) | .033 | 1.31  (0.49 – 2.13) | .003 | 0.22  (-1.56 – 2.01) | .798 |
| **Predictor: Emotional reactivity (decreased positive affect in response to stress)** | | | | | | | | |
| Outcome at baseline | 0.50  (0.15 – 0.85) | .005 | -0.13  (0.65 – 0.39) | .601 | 0.45  (0.18 – 0.73) | .002 | 0.56  (0.01 – 1.11) | .048 |
| Emotional reactivity | -0.97  (-2.01 – 0.07) | .065 | -0.99  (-2.38 – 0.40) | .152 | -1.09  (-1.96 –- 0.23) | .015 | -0.48  (-1.79 – 0.84) | .457 |
| **Predictor: Psychotic reactivity (increased psychotic experiences in response to stress)** | | | | | | | | |
| Outcome at baseline | 0.45  (0.09 – 0.82) | .016 | -0.20  (-0.73 – 0.34) | .450 | 0.34  (0.05 – 0.63) | .021 | 0.57  (0.05 – 1.09) | .033 |
| Psychotic reactivity | 0.80  (-0.31 – 1.92) | .150 | 1.41  (-0.35 – 3.17) | .111 | 1.09  (0.18 – 2.00) | .020 | 1.19  (-0.37 – 2.74) | .125 |
|  | **Anxiety (CAARMS)** | | | | **Tolerance to normal stress (CAARMS)** | | | |
|  | 1-year follow-up (*N*=39) | | 2-year follow-up (*N*=30) | | 1-year follow-up (*N*=39) | | 2-year follow-up (*N*=30) | |
|  | *B* (95% CI) | *P* | *B* (95% CI) | *P* | *B* (95% CI) | *P* | *B* (95% CI) | *P* |
| **Predictor: Emotional reactivity (increased negative affect in response to stress)** | | | | | | | | |
| Outcome at baseline | 0.31  (-0.19 – 0.81) | .222 | 0.85  (-0.31 – 2.02) | .141 | 0.29  (0.00 – 0.57) | .054 | 0.22  (-0.19 – 0.63) | .270 |
| Emotional reactivity | 0.030  (-0.56 – 1.17) | .478 | -0.73  (-2.52 – 1.06) | .404 | -0.03  (-0.97 – 0.91) | .950 | -0.20  (-1.80 – 1.39) | .794 |
| **Predictor: Emotional reactivity (decreased positive affect in response to stress)** | | | | | | | | |
| Outcome at baseline | 0.27  (-0.18 – 0.71) | .230 | 0.071  (-0.42 – 1.83) | .204 | 0.28  (-0.01 – 0.56) | .058 | 0.21  (-0.19 – 0.60) | .286 |
| Emotional reactivity | -0.81  (-1.58 – -0.03) | .041 | 0.10  (-1.24 – 1.45) | .873 | 0.36  (0.56 – 1.29) | .428 | 0.02  (-1.16 – 1.21) | .967 |
| **Predictor: Psychotic reactivity (increased psychotic experiences in response to stress)** | | | | | | | | |
| Outcome at baseline | 0.35  (-0.14 – 0.83) | .155 | 0.75  (-0.34 – 1.84) | .168 | 0.28  (-0.01 – 0.58) | .057 | 0.23  (-0.18 – 0.65) | .252 |
| Psychotic reactivity | 0.17  (-0.68 – 1.02) | .689 | -0.84  (-2.45 – 0.77) | .288 | 0.01  (-0.95 – 0.97) | .985 | -0.29  (-1.83 – 1.25) | .701 |

*Note*. Results adjusted for age, gender, ethnicity, centre, comorbid major depressive and anxiety disorders, and time to follow-up. Illness severity assessed with the Clinical Global Impression Scale (CGI). Level of functioning assessed with the Global Assessment of Functioning Scale (GAF). *N* = sample size, 95% CI = 95% confidence interval.

### *5.2.2 Emotional and psychotic stress reactivity as mediators of the association of childhood trauma and clinical outcomes (H4)*

As displayed in Table S6, the adjusted analysis in the restricted sample showed similar results compared to the main analysis. Increased negative affect in response to stress mediated the association of childhood trauma and illness severity at 1-year follow-up (indirect effect: *B*=0.19, 95% CI 0.02 – 0.37, *P*=.030). Moreover, increased psychotic experiences in response to stress mediated the association of childhood trauma and unusual thought content at 1-year follow-up (indirect effect: *B*=0.36, 95% CI 0.02 – 0.70, *P*=.037). In addition, the association of childhood trauma and perceptual abnormalities at 1-year follow-up was mediated by increased negative affect (indirect effect: *B*=0.40, 95% CI 0.08 – 0.72, *P*=.013) and increased psychotic experiences in response to stress (indirect effect: *B*=0.43, 95% CI 0.11 – 0.75, *P*=.008).

|  | **Clinical Outcomes** | | | | | | | |
| --- | --- | --- | --- | --- | --- | --- | --- | --- |
|  | **Illness severity (CGI)** | | | | **Level of functioning Disability (GAF)** | | | |
|  | 1-year follow-up (*N*=42) | | 2-year follow-up (*N*=32) | | 1-year follow-up (*N*=46) | | 2-year follow-up (*N*=30) | |
|  | *B* (95% CI) | *P* | *B* (95% CI) | *P* | *B* (95% CI) | *P* | *B* (95% CI) | *P* |
| **Mediator: Emotional reactivity (increased negative affect in response to stress)** | | | | | | | | |
| Total effect | 0.49 (0.15 – 0.83) | .004 | -0.48 (-0.95 – 0.00) | .052 | -3.00 (-7.12 – 1.12) | .153 | 2.93 (-2.55 – 8.40) | .295 |
| Direct effect | 0.30 (-0.02 – 0.62) | .068 | -0.58 (-1.12 - -0.04) | .034 | -1.80 (-6.02 – 2.42) | .403 | 2.89 (-3.38 – 9.17) | .366 |
| Indirect effect | 0.19 (0.02 – 0.37) | .030 | 0.11 (-0.10 – 0.31) | .305 | -1.20 (-3.02 – 0.61) | .195 | 0.03 (-2.31 – 2.38) | .978 |
| **Mediator: Emotional reactivity (decreased positive affect in response to stress)** | | | | | | | | |
| Total effect | 0.44 (0.12 – 0.77) | .007 | -0.45 (-0.90 – 0.01) | .054 | -2.82 (-6.83– 1.20) | .169 | 2.99 (-2.42 – 8.39) | .279 |
| Direct effect | 0.35 (0.05 – 0.66) | .024 | -0.53 (-0.98 – -0.08) | .021 | -1.99 (-5.92 – 1.94) | .321 | 3.19 (-2.31 – 8.69) | .637 |
| Indirect effect | 0.09 (-0.03 – 0.22) | .148 | 0.08 (-0.04 – 0.21) | .182 | -0.83 (-2.04 – 0.39) | .182 | -0.20 (-1.08 – 0.68) | .651 |
| **Mediator: Psychotic reactivity (increased psychotic experiences in response to stress)** | | | | | | | | |
| Total effect | 0.41 (0.07 – 0.75) | .018 | -0.43 (-0.91 – 0.05) | .078 | -2.73 (-6.88 – 1.43) | .198 | 3.41 (-2.03 – 8.86) | .219 |
| Direct effect | 0.30 (-0.03 – 0.64) | .075 | -0.49 (-1.01 – 0.02) | .059 | -2.61 (-6.96 – 1.74) | .240 | 2.03 (-3.55 – 7.61) | .476 |
| Indirect effect | 0.11 (-0.04 – 0.26) | .149 | 0.06 (-0.15 – 0.28) | .562 | -0.12 (-2.03 – 1.80) | .903 | 1.38 (-1.30 – 4.07) | .313 |
|  | **Unusual thought content (CAARMS)** | | | | **Perceptual abnormalities (CAARMS)** | | | |
|  | 1-year follow-up (*N*=39) | | 2-year follow-up (*N*=30) | | 1-year follow-up (*N*=39) | | 2-year follow-up (*N*=29) | |
|  | *B* (95% CI) | *P* | *B* (95% CI) | *P* | *B* (95% CI) | *P* | *B* (95% CI) | *P* |
| **Mediator: Emotional reactivity (increased negative affect in response to stress)** | | | | | | | | |
| Total effect | 0.04 (-0.61 – 0.69) | .909 | 0.45 (-0.38 – 1.27) | .287 | -0.03 (-0.58 – 0.51) | .900 | 0.00 (-0.90 – 0.91) | .993 |
| Direct effect | -0.26 (-0.92 – 0.39) | .430 | 0.17 (-0.73 – 1.06) | .717 | -0.44 (-0.94 – 0.07) | .090 | 0.25 (-0.75 – 1.24) | .628 |
| Indirect effect | 0.30 (-0.02 – 0.62) | .063 | 0.28 (-0.09 – 0.65) | .136 | 0.40 (0.08 – 0.72) | .013 | -0.24 (-0.64 – 0.15) | .229 |
| **Mediator: Emotional reactivity (decreased positive affect in response to stress)** | | | | | | | | |
| Total effect | 0.04 (-0.61 – 0.68) | .908 | 0.49 (-0.32 – 1.30) | .240 | -0.06 (-0.31 – 0.50) | .844 | -0.05 (-0.96 – 0.86) | .910 |
| Direct effect | -0.07 (-0.70 – 0.56) | .816 | 0.39 (-0.42 – 1.20) | .349 | -0.17 (-0.70 – 0.37) | .541 | -0.09 (-1.02 – 0.83) | .841 |
| Indirect effect | 0.11 (-0.06 – 0.29) | .209 | 0.10 (-0.07 – 0.26) | .245 | 0.11 (-0.05 – 0.28) | .184 | 0.04 (-0.10 – 0.18) | .553 |
| **Mediator: Psychotic reactivity (increased psychotic experiences in response to stress)** | | | | | | | | |
| Total effect | 0.09 (-0.55 – 0.74) | .780 | 0.60 (-0.23 – 1.44) | .157 | 0.02 (-0.52 – 0.56) | .936 | 0.03 (-0.90 – 0.96) | .950 |
| Direct effect | -0.27 (-0.91 -0.37) | .408 | 0.37 (-0.46 – 1.21) | .381 | -0.41 (-0.92 – 0.10) | .112 | -0.13 (-.106 – 0.80) | .782 |
| Indirect effect | 0.36 (0.02 – 0.70) | 0.37 | 0.23 (-0.13 – 0.59) | .213 | 0.43 (0.11 – 0.75) | .008 | 0.16 (-0.23 – 0.56) | .426 |
|  | **Anxiety (CAARMS)** | | | | **Tolerance to normal stress (CAARMS)** | | | |
|  | 1-year follow-up (*N*=39) | | 2-year follow-up (*N*=30) | | 1-year follow-up (*N*=39) | | 2-year follow-up (*N*=30) | |
|  | *B* (95% CI) | *P* | *B* (95% CI) | *P* | *B* (95% CI) | *P* | *B* (95% CI) | *P* |
| **Mediator: Emotional reactivity (increased negative affect in response to stress)** | | | | | | | | |
| Total effect | -0.19 (-0.63 – 0.25) | .389 | -0.29 (-1.14 – 0.56) | .499 | -0.15 (-0.68 – 0.38) | .576 | -0.11 (-0.83 – 0.61) | .767 |
| Direct effect | -0.38 (-0.82 – 0.07) | .099 | -0.20 (-1.15 – 0.74) | .670 | -0.18 (-0.74 – 0.37) | .513 | -0.14 (-0.94 – 0.66) | .730 |
| Indirect effect | 0.18 (-0.03 – 0.39) | .087 | -0.09 (-0.44 – 0.26) | .620 | 0.03 (-0.18 – 0.25) | .759 | 0.03 (-0.26 – 0.33) | .830 |
| **Mediator: Emotional reactivity (decreased positive affect in response to stress)** | | | | | | | | |
| Total effect | -0.18 (-0.61 – 0.25) | .415 | -0.31 (-1.16 – 0.54) | .472 | -0.18 (-0.70 – 0.35) | .509 | -0.11 (-0.83 – 0.61) | .774 |
| Direct effect | -0.28 (-0.69 – 0.14) | .190 | -0.32 (-1.18 – 0.54) | .470 | -0.14 (-0.66 – 0.38) | .602 | -0.12 (-0.85 – 0.61) | .742 |
| Indirect effect | 0.10 (-0.04 – 0.24) | .168 | 0.01 (-0.11 – 0.13) | .921 | -0.04 (0.14 – 0.06) | .472 | 0.02 (-0.09 – 0.12) | .752 |
| **Mediator: Psychotic reactivity (increased psychotic experiences in response to stress)** | | | | | | | | |
| Total effect | -0.19 (-0.63 – 0.26) | .414 | -0.41 (-1.26 – 0.44) | .349 | -0.13 (-0.66 – 0.39) | .618 | -0.10 (-0.83 – 0.64) | .794 |
| Direct effect | -0.32 (-0.78 – 0.13) | .164 | -0.19 (-1.04 – 0.66) | .661 | -0.21 (-0.75 – 0.34) | .462 | -0.11 (-0.85 – 0.63) | .772 |
| Indirect effect | 0.14 (-0.07 – 0.35) | .204 | -0.22 (-0.58 – 0.15) | .246 | 0.07 (-0.17 – 0.31) | .568 | 0.01 (-0.29 – 0.31) | .937 |

**Table S6.** Restricted sample: Emotional and psychotic stress reactivity as mediators of the association of childhood trauma and clinical outcomes

*Note*. Results adjusted for age, gender, ethnicity, centre, comorbid major depressive and anxiety disorders, and time to follow-up. Childhood trauma assessed with the CTQ. Illness severity assessed with the Clinical Global Impression Scale (CGI). Level of functioning assessed with the Global Assessment of Functioning Scale (GAF). Unusual thought content, perceptual abnormalities, anxiety and tolerance to normal stress assessed with the Comprehensive Assessment of At Risk Mental State (CAARMS). *N* = sample size, 95% CI = 95% confidence interval.

# **Unadjusted analysis in the full sample**

## **6.1 Method**

In Supplementary Material 6, we report the results of the unadjusted analyses in the full sample.

## **6.2 Results**

### *6.2.1 Association between momentary stress, affect and psychotic experiences (H1)*

Momentary stress was associated with small to moderate increases in negative affect (negative affect; β=0.31, 95% CI 0.27 – 0.36, *P*<.001) and psychotic experiences (psychotic experiences; β=0.16, 95% CI 0.13 – 0.19, *P*<.001) as well as with a moderate decrease in positive affect (positive affect; β=-0.39, 95% CI -0.43 – -0.34, *P*<.001).

### *6.2.2 Association between momentary stress, affect and psychotic experiences by childhood trauma (H2)*

As displayed in Table S7, childhood trauma modified the associations of momentary stress and negative affect (stress × childhood trauma: β=0.03, 95% CI 0.01 – 0.06, *P*=.018) and psychotic experiences (stress × childhood trauma: β=0.02, 95% CI 0.00 – 0.05, *P*=.037). These associations were greater in individuals with high levels of childhood trauma (outcome negative affect: high vs. low childhood trauma: β=0.06, 95% CI 0.01 – 0.11, *P*=.018; outcome psychotic experiences: high vs. low childhood trauma: β=0.05, 95% CI 0.00 – 0.09, *P*=.037). The results are congruent with the adjusted analysis.

**Table S7.** Modification of the association between momentary stress and affect/psychotic experiences by childhood trauma

| **Effect modification by childhood trauma** | | | | |
| --- | --- | --- | --- | --- |
|  | β | 95% CI | SE | *P* |
| **Outcome: Negative affect** |  |  |  |  |
| Stress | 0.31 | 0.28 – 0.34 | 0.01 | <.001 |
| Childhood trauma | 0.26 | 0.12 – 0.40 | 0.07 | <.001 |
| Stress × childhood trauma | 0.03 | 0.01 – 0.06 | 0.01 | .018 |
| High childhood trauma | 0.34 | 0.31 – 0.37 | 0.02 | <.001 |
| Low childhood trauma | 0.28 | 0.24 – 0.32 | 0.02 | <.001 |
| High vs. low childhood trauma | 0.06 | 0.01 – 0.11 | 0.03 | .018 |
| **Outcome: Positive affect** |  |  |  |  |
| Stress | -0.39 | -0.42 – -0.36 | 0.02 | <.001 |
| Childhood trauma | -0.16 | -0.28 – -0.03 | 0.07 | .014 |
| Stress × childhood trauma | 0.03 | 0.00 – 0.06 | 0.01 | .084 |
| **Outcome: Psychotic experiences** |  |  |  |  |
| Stress | 0.15 | 0.13 – 0.17 | 0.01 | <.001 |
| Childhood trauma | 0.31 | 0.17 – 0.46 | 0.07 | <.001 |
| Stress × childhood trauma | 0.02 | 0.00 – 0.05 | 0.01 | .037 |
| High childhood trauma | 0.17 | 0.14 – 0.20 | 0.02 | <.001 |
| Low childhood trauma | 0.12 | 0.09 – 0.16 | 0.02 | <.001 |
| High vs. low childhood trauma | 0.05 | 0.00 – 0.09 | 0.02 | .037 |

*Note*. Childhood trauma assessed with the CTQ. 95% CI = 95% confidence interval, SE = standard error.

### *6.2.3 Stress reactivity and clinical outcomes at follow-up (H3)*

Table S8 shows the unadjusted results on the association of emotional and psychotic stress reactivity with illness severity and level of functioning at 1- and 2-year follow-up. Illness severity at 1-year follow-up was predicted by increased negative affect in response to stress (*B*=0.55, 95% CI 0.03 – 1.06, *P*=.037). Level of functioning at 1-year follow-up was predicted by decreased positive affect in response to stress (*B*=7.64, 95%CI 1.82– 13.46, *P*=.011). In addition, increased negative affect (*B*=1.31, 95%CI 0.72 – 1.90, *P<*.001) and increased psychotic experiences (*B*=1.00, 95%CI 0.35 – 1.66, *P*=.004) in response to stress predicted perceptual abnormalities at 1-year follow-up. There was no evidence that emotional or psychotic stress reactivity predicted unusual thought content, anxiety or tolerance to normal stress at follow-up.

**Table S8.** Clinical Outcomes at 1- and 2-year follow-up predicted by emotional and psychotic stress reactivity at baseline and clinical outcome at baseline

|  | **Clinical Outcomes** | | | | | | | |
| --- | --- | --- | --- | --- | --- | --- | --- | --- |
|  | **Illness severity (CGI)** | | | | **Level of functioning Disability (GAF)** | | | |
|  | 1-year follow-up (*N*=47) | | 2-year follow-up (*N*=36) | | 1-year follow-up (*N*=48) | | 2-year follow-up (*N*=36) | |
|  | *B* (95% CI) | *P* | *B* (95% CI) | *P* | *B* (95% CI) | *P* | *B* (95% CI) | *P* |
| **Predictor: Emotional reactivity (increased negative affect in response to stress)** | | | | | | | | |
| Outcome at baseline | 0.63  (0.37 – 0.88) | <.001 | 0.46  (0.11 – 0.81) | .012 | 0.42  (0.15 – 0.70) | .003 | 0.37  (-0.02 – 0.76) | .059 |
| Emotional reactivity | 0.55  (0.03 – 1.06) | .037 | -0.01  (-0.61 – 0.59) | .981 | -5.82  (-11.64 – -0.01) | .050 | 0.66  (-6.07 –7.39) | .844 |
| **Predictor: Emotional reactivity (decreased positive affect in response to stress)** | | | | | | | | |
| Outcome at baseline | 0.62  (0.35 – 0.89) | <.001 | 0.36  (0.01 – 0.71) | .043 | 0.39  (0.12 – 0.66) | .006 | 0.39  (0.01 – 0.77) | .045 |
| Emotional reactivity | -0.43  (-0.95 – -0.10) | .108 | -0.56  (-1.21 – 0.10) | .092 | 7.64  (1.82 – 13.46) | .011 | 3.61  (-3.53 – 10.75) | .311 |
| **Predictor: Psychotic reactivity (increased psychotic experiences in response to stress)** | | | | | | | | |
| Outcome at baseline | 0.67  (0.40 – 0.93) | <.001 | 0.47  (0.12 – 0.81) | .010 | 0.44  (0.15 – 0.72) | .003 | 0.35  (-0.05 – 0.75) | .080 |
| Psychotic reactivity | 0.22  (-0.28 – 0.73) | .377 | -0.17  (-0.80 – 0.45) | .578 | -4.11  (-9.81 – 1.60) | .154 | 1.60  (-5.62 – 8.81) | .655 |
|  | **Unusual thought content (CAARMS)** | | | | **Perceptual abnormalities (CAARMS)** | | | |
|  | 1-year follow-up (*N*=44) | | 2-year follow-up (*N*=34) | | 1-year follow-up (*N*=44) | | 2-year follow-up (*N*=34) | |
|  | *B* (95% CI) | *P* | *B* (95% CI) | *P* | *B* (95% CI) | *P* | *B* (95% CI) | *P* |
| **Predictor: Emotional reactivity (increased negative affect in response to stress)** | | | | | | | | |
| Outcome at baseline | 0.48  (0.19 – 0.77) | .002 | 0.14  (-0.26 – 0.54) | .477 | 0.42  (0.18 – 0.65) | .001 | 0.36  (-0.05 – 0.77) | .082 |
| Emotional reactivity | 0.49  (-0.32 – 1.30) | .229 | 0.43  (-0.76 – 1.61) | .470 | 1.31  (0.72 – 1.90) | <.001 | -0.18  (-1.24 – 0.87) | .724 |
| **Predictor: Emotional reactivity (decreased positive affect in response to stress)** | | | | | | | | |
| Outcome at baseline | 0.50  (0.22 – 0.79) | .001 | 0.19  (-0.19 – 0.73) | .320 | 0.48  (0.20 – 0.75) | .001 | 0.44  (-0.02 – 0.86) | .042 |
| Emotional reactivity | -0.64  (-1.52 – 0.24) | .152 | -0.46  (-1.65 – 0.73) | .435 | -0.74  (-1.50 – -0.01) | .054 | -0.65  (-1.76 – 0.47) | .248 |
| **Predictor: Psychotic reactivity (increased psychotic experiences in response to stress)** | | | | | | | | |
| Outcome at baseline | 0.45  (0.16 –0.74) | .003 | 0.07  (-0.32 – 0.47) | .703 | 0.36  (0.10 – 0.62) | .009 | 0.34  (-0.07 – 0.74) | .101 |
| Psychotic reactivity | 0.46  (-0.35 – 1.28) | .257 | 0.98  (-0.26 – 2.21) | .116 | 1.00  (0.35 – 1.66) | .004 | 0.53  (-0.56 – 1.63) | .328 |
|  | **Anxiety (CAARMS)** | | | | **Tolerance to normal stress (CAARMS)** | | | |
|  | 1-year follow-up (*N*=44) | | 2-year follow-up (*N*=34) | | 1-year follow-up (*N*=44) | | 2-year follow-up (*N*=34) | |
|  | *B* (95% CI) | *P* | *B* (95% CI) | *P* | *B* (95% CI) | *P* | *B* (95% CI) | *P* |
| **Predictor: Emotional reactivity (increased negative affect in response to stress)** | | | | | | | | |
| Outcome at baseline | 0.38  (-0.02 – 0.79) | .063 | 0.72  (-0.07 – 1.52) | .074 | 0.34  (0.10 – 0.58) | .007 | 0.10  (-0.24 – 0.44) | .555 |
| Emotional reactivity | 0.30  (-0.38 – 0.97) | .384 | -0.39  (-1.53 – 0.75) | .494 | 0.00  (-0.67 – 0.66) | .995 | -0.02  (-1.05 – 1.01) | .974 |
| **Predictor: Emotional reactivity (decreased positive affect in response to stress)** | | | | | | | | |
| Outcome at baseline | 0.37  (-0.01 – 0.76) | .057 | 0.61  (-0.12 – 1.34) | .097 | 0.34  (0.10– 0.58) | .007 | 0.08  (-0.23 – 0.39) | .606 |
| Emotional reactivity | -0.61  (-1.31 – 0.10) | .090 | -0.20  (-1.29 – 0.88) | .706 | 0.01  (-0.71 – 0.73) | .971 | -0.67  (-1.64 – 0.30) | .168 |
| **Predictor: Psychotic reactivity (increased psychotic experiences in response to stress)** | | | | | | | | |
| Outcome at baseline | 0.41  (0.02 – 0.80) | .039 | 0.63  (-0.07 – 1.33) | .078 | 0.34  (0.10 – 0.59) | .007 | 0.12  (-0.20 – 0.45) | .454 |
| Psychotic reactivity | 0.37  (-0.28 – 1.01) | .255 | -0.81  (-1.86 – 0.25) | .129 | -0.03  (-0.69 – 0.63) | .932 | -0.32  (-1.35– 0.70) | .522 |

*Note*. Illness severity assessed with the Clinical Global Impression Scale. Level of functioning assessed with the Global Assessment of Functioning Scale.

### *6.2.4 Emotional and psychotic stress reactivity as mediators of the association of childhood trauma and clinical outcomes (H4)*

Table S9 shows unadjusted findings on total, direct, and indirect effects of childhood trauma, emotional and psychotic stress reactivity on illness severity and level of functioning at follow-up. Increased negative affect in response to stress mediated the effect of childhood trauma on illness severity at 1-year follow-up (indirect effect: *B*=0.23, 95% CI 0.02 – 0.31, *P*=.030). Decreased positive affect in response to stress mediated the effect of childhood trauma on illness severity at 2-year follow-up (indirect effect: *B*=0.17, 95% CI 0.01 – 0.34, *P*=.039). Higher levels of childhood trauma were associated with more intense emotional stress reactivity in form of a stronger reduction of positive affect and an increase of negative affect when exposed to momentary stress. Stronger reduction of positive affect and stronger increase of negative affect in response to stress, in turn, were associated with higher ratings of illness severity at follow-up. Furthermore, psychotic reactivity to stress mediated the effect of childhood trauma on unusual thought content at 1-year follow-up (indirect effect: *B*=0.32, 95% CI 0.02 – 0.62, *P*=.037). In addition, the association of childhood trauma and perceptual abnormalities at 1-year follow-up was mediated by increased negative affect up (indirect effect: *B*=0.44, 95% CI 0.15 – 0.73, *P*=.003) and increased psychotic experiences up (indirect effect: *B*=0.44, 95% CI 0.16 – 0.73, *P*=.002) in response to stress.

|  | **Clinical Outcomes** | | | | | | | |
| --- | --- | --- | --- | --- | --- | --- | --- | --- |
|  | **Illness severity (CGI)** | | | | **Level of functioning Disability (GAF)** | | | |
|  | 1-year follow-up (*N*=48) | | 2-year follow-up (*N*=37) | | 1-year follow-up (*N*=48) | | 2-year follow-up (*N*=35) | |
|  | *B* (95% CI) | *P* | *B* (95% CI) | *P* | *B* (95% CI) | *P* | *B* (95% CI) | *P* |
| **Mediator: Emotional reactivity (increased negative affect in response to stress)** | | | | | | | | |
| Total effect | 0.45 (0.07 – 0.83) | .022 | -0.11 (-0.59 – 0.37) | .644 | -3.83 (-7.56 – -0.09) | .045 | 1.65 (-3.58– 6.88) | .536 |
| Direct effect | 0.22 (-0.13 – 0.57) | .222 | -0.16 (-0.64 – -0.33) | .527 | -2.64 (-6.39– 1.10) | .166 | 1.38 (-3.99 – 6.74) | .615 |
| Indirect effect | 0.23 (0.02 – 0.43) | .030 | 0.04 (-0.13 – 0.22) | .622 | -1.19 (-3.02 – 0.64) | .202 | 0.28 (-1.62– 2.17) | .776 |
| **Mediator: Emotional reactivity (decreased positive affect in response to stress)** | | | | | | | | |
| Total effect | 0.36 (0.00 – 0.73) | .051 | -0.13 (-0.57 – 0.32) | .575 | -3.82 (-7.45– -0.19) | .039 | 1.65 (-3.50 – 6.79) | .531 |
| Direct effect | 0.22 (-0.14 – -0.57) | .234 | -0.30 (-0.74 – -0.15) | .187 | -2.41 (-5.97– 1.14) | .184 | 2.43 (-2.91 – 7.77) | .373 |
| Indirect effect | 0.15 (0.00 – 0.30) | .052 | 0.17 (0.01 – 0.34) | .039 | -1.41 (-2.89 – 0.08) | .063 | -0.78 (-2.26 – 0.70) | .302 |
| **Mediator: Psychotic reactivity (increased psychotic experiences in response to stress)** | | | | | | | | |
| Total effect | 0.37 (-0.01 – 0.75) | .060 | -0.13 (-0.62 – 0.35) | .091 | -3.45 (-7.18– 0.28) | .070 | 1.89 (-3.34 – 7.11) | .479 |
| Direct effect | 0.24 (-0.14 – 0.62) | .214 | -0.12 (-0.62 – 0.37) | .619 | -2.72 (-6.92 – 1.48) | .205 | 0.97 (-4.38 – 6.32) | .723 |
| Indirect effect | 0.13 (-0.07 – 0.32) | .211 | -0.01 (-0.21 – 0.19) | .928 | -0.43 (-2.33 – 1.47) | .658 | 0.92 (-1.29 – 3.13) | .415 |
|  | **Unusual thought content (CAARMS)** | | | | **Perceptual abnormalities (CAARMS)** | | | |
|  | 1-year follow-up (*N*=45) | | 2-year follow-up (*N*=34) | | 1-year follow-up (*N*=45) | | 2-year follow-up (*N*=33) | |
|  | *B* (95% CI) | *P* | *B* (95% CI) | *P* | *B* (95% CI) | *P* | *B* (95% CI) | *P* |
| **Mediator: Emotional reactivity (increased negative affect in response to stress)** | | | | | | | | |
| Total effect | -0.17 (-0.70 – 0.36) | .534 | 0.65 (-0.22 – 1.52) | .146 | 0.11 (-0.33 – 0.55) | .621 | -0.09 (-0.94 – 0.75) | .830 |
| Direct effect | -0.38 (-0.93 – 0.18) | .181 | 0.50 (-0.32– 1.31) | .235 | -0.33 (0.73 – 0.08) | .116 | -0.03 (-0.82 – 0.77) | .951 |
| Indirect effect | 0.21 (-0.06 – 0.48) | .123 | 0.15 (-0.15 – 0.46) | .318 | 0.44 (0.15 – 0.73) | .003 | -0.07 (-0.36– 0.22) | .645 |
| **Mediator: Emotional reactivity (decreased positive affect in response to stress)** | | | | | | | | |
| Total effect | -0.17 (-0.70 – 0.36) | .539 | 0.53 (-0.30 – 1.37) | .210 | 0.07 (-0.39 – 0.54) | .754 | -0.01 (-0.79 – 0.81) | .980 |
| Direct effect | -0.28 (-0.82 – 0.26) | .305 | 0.47 (-0.36 – 1.30) | .272 | -0.04 (-0.51 – 0.43) | .875 | -0.05 (-0.85 – 0.75) | .907 |
| Indirect effect | 0.11 (-0.08 – 0.31) | .255 | 0.07 (-0.15 – 0.28) | .526 | 0.11 (-0.06 – 0.29) | .211 | 0.06 (-0.15 – 0.26) | .582 |
| **Mediator: Psychotic reactivity (increased psychotic experiences in response to stress)** | | | | | | | | |
| Total effect | -0.17 (-0.69 – 0.35) | .523 | 0.75 (-0.10 – 1.59) | .084 | 0.08 (-0.36 – 0.53) | .709 | 0.14 (-0.68 – 0.97) | .734 |
| Direct effect | -0.49(-1.04 – 0.07) | .084 | 0.43 (-0.36 – 1.22) | .287 | -0.36 (-0.79 – 0.0708 | .106 | -0.06 (-0.85 – 0.72) | .872 |
| Indirect effect | 0.32 (0.02– 0.62) | .037 | 0.31 (-0.04 – 0.67) | .084 | 0.44 (0.16 – 0.73) | .002 | 0.21 (-0.13 – 0.54) | .228 |
|  | **Anxiety (CAARMS)** | | | | **Tolerance to normal stress (CAARMS)** | | | |
|  | 1-year follow-up (*N*=45) | | 2-year follow-up (*N*=34) | | 1-year follow-up (*N*=45) | | 2-year follow-up (*N*=34) | |
|  | *B* (95% CI) | *P* | *B* (95% CI) | *P* | *B* (95% CI) | *P* | *B* (95% CI) | *P* |
| **Mediator: Emotional reactivity (increased negative affect in response to stress)** | | | | | | | | |
| Total effect | -0.16 (-0.24 – 0.56) | .432 | -0.36 (-1.20 – 0.47) | .391 | 0.04 (-0.40 – 0.47) | .873 | 0.19 (-0.55 – 0.93) | .618 |
| Direct effect | -0.04 (-0.38 – 0.46) | .860 | -0.38 (-1.16 – 0.41) | .345 | -0.05 (-0.51 – 0.41) | .840 | 0.16 (-0.54 – 0.86) | .649 |
| Indirect effect | 0.12 (-0.07 – 0.32) | .224 | 0.01 (-0.27 – 0.29) | .930 | 0.08 (-0.13 – 0.29) | .435 | 0.03 (-0.22 – 0.28) | .830 |
| **Mediator: Emotional reactivity (decreased positive affect in response to stress)** | | | | | | | | |
| Total effect | 0.19 (-0.21 – 0.56) | .366 | -0.35 (-1.14 – 0.44) | .388 | 0.02 (-0.41 – 0.45) | .928 | 0.24 (-0.45 – 0.93) | .494 |
| Direct effect | 0.05 (-0.35 – 0.44) | .821 | -0.40 (-1.18– 0.39) | .322 | 0.03 (-0.42 – 0.47) | .911 | 0.11 (-0.57 – 0.79) | .751 |
| Indirect effect | 0.14 (-0.02 – 0.30) | .096 | 0.05 (-0.15 – 0.25) | .639 | -0.01 (-0.16 – 0.15) | .944 | 0.13 (-0.06 – 0.32) | .184 |
| **Mediator: Psychotic reactivity (increased psychotic experiences in response to stress)** | | | | | | | | |
| Total effect | 0.15 (-0.25 – 0.55) | .332 | -0.56 (-1.37 – 0.25) | .176 | 0.03 (-0.40 – 0.46) | .884 | 0.10 (-0.64 – 0.83) | .799 |
| Direct effect | 0.03 (-0.40 – 0.47) | .887 | -0.33 (-1.09 – 0.43) | .394 | -0.07 (-0.55 – 0.40) | .763 | 0.18 (-0.52– 0.87) | .618 |
| Indirect effect | 0.12 (-0.09 – 0.33) | .269 | -0.23 (-0.56 – 0.10) | .173 | 0.10 (-0.12 – 0.33) | .370 | -0.08 (-0.37 – 0.21) | .581 |

**Table S9.** Emotional and psychotic stress reactivity as mediators of the association of childhood trauma and clinical outcomes

*Note*. Illness severity assessed with the Clinical Global Impression Scale. Level of functioning assessed with the Global Assessment of Functioning Scale.

# **Exploratory analyses: The role of transition**

## **7.1 Does transition moderate the effect of momentary stress on affect and psychotic experiences?**

### *7.1.1 Method*

As ESM data have a multilevel structure with multiple observations (level-1) nested within participants (level-2), the ‘mixed’ command in Stata 15 was used to fit two-level, linear mixed models (StataCorp, 2017). The composite stress measure and transition status were included as independent variables, and negative affect, positive affect, and psychotic experiences as outcome variables. To examine effect modification by transition, we included two-way interaction-terms for stress × transition. Analyses were performed twice, once unadjusted, once while controlling for potential confounders (i.e., age, gender, centre, ethnicity, comorbid major depressive and anxiety disorders).

### *7.1.2 Results*

Table S10 shows the results of the adjusted analyses. Transition status modified the associations of momentary stress with negative affect (stress × transition: β=0.11, 95% CI 0.02 – 0.20, *P*=.021) and positive affect (stress × transition: β=-0.14, 95% CI -0.25 – -0.03, *P*=.010). These associations were greater in individuals who transitioned to psychosis (outcome negative affect: transition vs. non-transition: β=0.11, 95% CI 0.02 – 0.20, *P=*.021; outcome positive affect: transition vs. non-transition: β=-0.14, 95% CI -0.24 – -0.03, *P*=.010). However, transition status did not modify the effect of momentary stress on psychotic experiences (stress × transition: β=0.01, 95% CI -0.07 – 0.09, *P*=.814).

Table 11S shows the results of the unadjusted analyses. Transition modified the associations of momentary stress with negative affect (stress × transition: β=0.11, 95% CI 0.02 – 0.20, *P*=.021) and positive affect (stress × transition: β=-0.14, 95% CI -0.24 – -0.03, *P*=.011). The associations were greater in individuals, who transitioned to psychosis compared to those who did not transition (outcome negative affect: transition vs. non-transition: β=0.11, 95% CI 0.02 – 0.20, *P=*.021; outcome positive affect: transition vs. non-transition: β=-0.14, 95% CI -0.24 – -0.03, *P*=.011). Adjusted and unadjusted results converge.

**Table S10.** Modification of the association between momentary stress and affect/psychotic experiences by transition status

| **Effect modification by transition status** | | | | |
| --- | --- | --- | --- | --- |
|  | β | 95% CI | SE | *P* |
| **Outcome: Negative affect** |  |  |  |  |
| Stress | 0.30 | 0.28 – 0.33 | 0.01 | <.001 |
| Transition status | 0.19 | -0.27 – 0.64 | 0.23 | .422 |
| Stress × transition status | 0.11 | 0.02 – 0.20 | 0.05 | .021 |
| Transition | 0.41 | 0.32 – 0.50 | 0.04 | <.001 |
| Non-transition | 0.30 | 0.28 – 0.33 | 0.01 | <.001 |
| Transition vs. non-transition | 0.11 | 0.02 – 0.20 | 0.05 | .021 |
| **Outcome: Positive affect** |  |  |  |  |
| Stress | -0.37 | -0.40 – -0.34 | 0.02 | <.001 |
| Transition status | -0.05 | -0.44 – 0.33 | 0.20 | .784 |
| Stress × transition status | -0.14 | -0.25 – -0.03 | 0.05 | .010 |
| Transition | -0.51 | -0.61 – -0.41 | 0.05 | <.001 |
| Non-transition | -0.37 | -0.40 – -0.34 | 0.02 | <.001 |
| Transition vs. non-transition | -0.14 | -0.24 – -0.03 | 0.05 | .010 |
| **Outcome: Psychotic experiences** |  |  |  |  |
| Stress | 0.16 | 0.13 – 0.18 | 0.01 | <.001 |
| Transition status | -0.11 | -0.63 – 0.40 | 0.26 | .668 |
| Stress × transition status | 0.01 | -0.07 – 0.09 | 0.04 | .814 |

*Note*. Results adjusted for age, gender, ethnicity, centre, comorbid major depressive and anxiety disorders. 95% CI = 95% confidence interval, SE = standard error.

**Table S11.** Modification of the association between momentary stress and affect/psychotic experiences by transition status (unadjusted)

| **Effect modification by transition status** | | | | |
| --- | --- | --- | --- | --- |
|  | β | 95% CI | SE | *P* |
| **Outcome: Negative affect** |  |  |  |  |
| Stress | 0.30 | 0.28 – 0.33 | 0.01 | <.001 |
| Transition status | 0.30 | -0.16 – 0.77 | 0.24 | .204 |
| Stress × transition status | 0.11 | 0.02 – 0.20 | 0.05 | .021 |
| Transition | 0.41 | 0.32 – 0.50 | 0.04 | <.001 |
| Non-transition | 0.30 | 0.28 – 0.33 | 0.01 | <.001 |
| Transition vs. non-transition | 0.11 | 0.02 – 0.20 | 0.05 | .021 |
| **Outcome: Positive affect** |  |  |  |  |
| Stress | -0.37 | -0.40 – -0.34 | 0.02 | <.001 |
| Transition status | -0.12 | -0.52 – 0.28 | 0.21 | .552 |
| Stress × transition status | -0.14 | -0.25 – -0.03 | 0.05 | .011 |
| Transition | -0.51 | -0.61 – -0.41 | 0.05 | <.001 |
| Non-transition | -0.37 | -0.40 – -0.34 | 0.02 | <.001 |
| Transition vs. non-transition | -0.14 | -0.24 – -0.03 | 0.05 | .011 |
| **Outcome: Psychotic experiences** |  |  |  |  |
| Stress | 0.15 | 0.13 – 0.18 | 0.01 | <.001 |
| Transition status | 0.16 | -0.37 – 0.70 | 0.27 | .549 |
| Stress × transition status | 0.01 | -0.07 – 0.09 | 0.04 | .790 |

*Note*. 95% CI = 95% confidence interval, SE = standard error.

## **7.2 Do emotional and psychotic stress reactivity mediate the association of childhood trauma and transition?**

### *7.2.1 Method*

To examine whether emotional and psychotic stress reactivity mediate the association of childhood trauma and transition, we used fitted values of psychotic experiences and affect predicted by the composite stress measure. We performed mediation analyses using the ‘gsem’ command. The total effect of childhood trauma on transition was apportioned into a direct effect and an indirect effect through stress reactivity. The indirect effect was computed using the product of coefficients strategy. The indirect and the total effect were computed and tested on significance using the ‘nlcom’ command. For transition, a Weibull distribution was assumed. Again, analyses were performed with and without adjusting for potential confounders.

### *7.2.2 Results*

As displayed in Tables S12 and S13, we found no evidence for direct effects of childhood trauma on time to transition and no evidence for mediation via emotional or psychotic stress reactivity.

**Table S12.** Emotional and psychotic stress reactivity as mediators of the association of childhood trauma and time to transition

|  | **Transition** (*N*=56) | | |  |  |  |  | |  |
| --- | --- | --- | --- | --- | --- | --- | --- | --- | --- |
|  | *B* (95% CI) | *P* |  |  |  |  |  | |  |
| **Mediator: Emotional reactivity (increased negative affect in response to stress)** | | | | | | | |  |  |
| Total effect | 0.90 (-0.21 – 2.01) | .112 |  |  |  |  |  | |  |
| Direct effect | 0.84 (-0.34 – 2.02) | .165 |  |  |  |  |  | |  |
| Indirect effect | 0.07 (-0.21 – 0.45) | .731 |  |  |  |  |  | |  |
| **Mediator: Emotional reactivity (decreased positive affect in response to stress)** | | | | | | | |  |  |
| Total effect | 0.94 (-0.16 – 2.04) | .093 |  |  |  |  |  | |  |
| Direct effect | 0.88 (-0.21 – 1.09) | .529 |  |  |  |  |  | |  |
| Indirect effect | 0.06 (-0.14 – 0.27) | .554 |  |  |  |  |  | |  |
| **Mediator: Psychotic reactivity (increased psychotic experiences in response to stress)** | | | | | | | |  |  |
| Total effect | 1.19 (-0.13 – 2.51) | .077 |  |  |  |  |  | |  |
| Direct effect | -0.76 (-2.56 – 1.04) | .408 |  |  |  |  |  | |  |
| Indirect effect | -0.22 (-0.76 – 0.32) | .418 |  |  |  |  |  | |  |

*Note*. Results adjusted for age, gender, ethnicity, centre, comorbid major depressive and anxiety disorders and time to follow-up. Childhood
trauma assessed with the CTQ. ). *N* = sample size, 95% CI = 95% confidence interval.

**Table S13.** Emotional and psychotic stress reactivity as mediators of the association of childhood trauma and time to transition (unadjusted analyses)

|  | **Transition** (*N*=56) | | |  |  |  |  | |  |
| --- | --- | --- | --- | --- | --- | --- | --- | --- | --- |
|  | *B* (95% CI) | *P* |  |  |  |  |  | |  |
| **Mediator: Emotional reactivity (increased negative affect in response to stress)** | | | | | | | |  |  |
| Total effect | 0.90 (-0.21 – 2.01) | .112 |  |  |  |  |  | |  |
| Direct effect | 0.84 (-0.34 – 2.02) | .165 |  |  |  |  |  | |  |
| Indirect effect | 0.07 (-0.21 – 0.45) | .731 |  |  |  |  |  | |  |
| **Mediator: Emotional reactivity (decreased positive affect in response to stress)** | | | | | | | |  |  |
| Total effect | 0.94 (-0.16 – 2.04) | .093 |  |  |  |  |  | |  |
| Direct effect | 0.88 (-0.21 – 1.09) | .529 |  |  |  |  |  | |  |
| Indirect effect | 0.06 (-0.14 – 0.27) | .554 |  |  |  |  |  | |  |
| **Mediator: Psychotic reactivity (increased psychotic experiences in response to stress)** | | | | | | | |  |  |
| Total effect | 1.19 (-0.13 – 2.51) | .077 |  |  |  |  |  | |  |
| Direct effect | -0.76 (-2.56 – 1.04) | .408 |  |  |  |  |  | |  |
| Indirect effect | -0.22 (-0.76 – 0.32) | .418 |  |  |  |  |  | |  |

*Note*. Childhood trauma assessed with the CTQ. ). *N* = sample size, 95% CI = 95% confidence interval.

# **Examining the structural validity of the ESM items**

## **8.1 Method**

To examine the structural validity of the ESM items, we conducted multilevel confirmatory factor analysis in R (R Core Team, 2019). We compared two models:

1. a model with a single ESM factor (Model A)
2. a model with correlated factors for negative affect, positive affect, psychotic experiences stress (Model B)

We compared the extent to which these models match the data using indicators of comparative model fit (information criteria, log-likelihood; Brown and Moore, 2012; Dziak *et al.*, 2020).

## **8.2 Results**

Table S14 gives an overview of the relevant indicators of comparative model fit. We found a better model fit for Model B, suggesting that this model with correlated factors of negative affect, positive affect and psychotic experiences matches the data better than a single ESM factor (Model A). Figures S2-S5 display detailed results of the multilevel confirmatory factor analyses.

**Table S14**. Model fit criteria

| **Model** | **AIC** | **BIC** | **ABIC** | **Log Likelihood** |
| --- | --- | --- | --- | --- |
| A | 154432.611 | 154911.231 | 154657.042 | -77136.305 |
| B | 151928.040 | 152442.557 | 152169.303 | -75878.020 |

*Note*. AIC = Akaike Information Criterion, BIC = Bayesian Information Criterion, ABIC = sample-size adjusted Bayesian
Information Criterion.


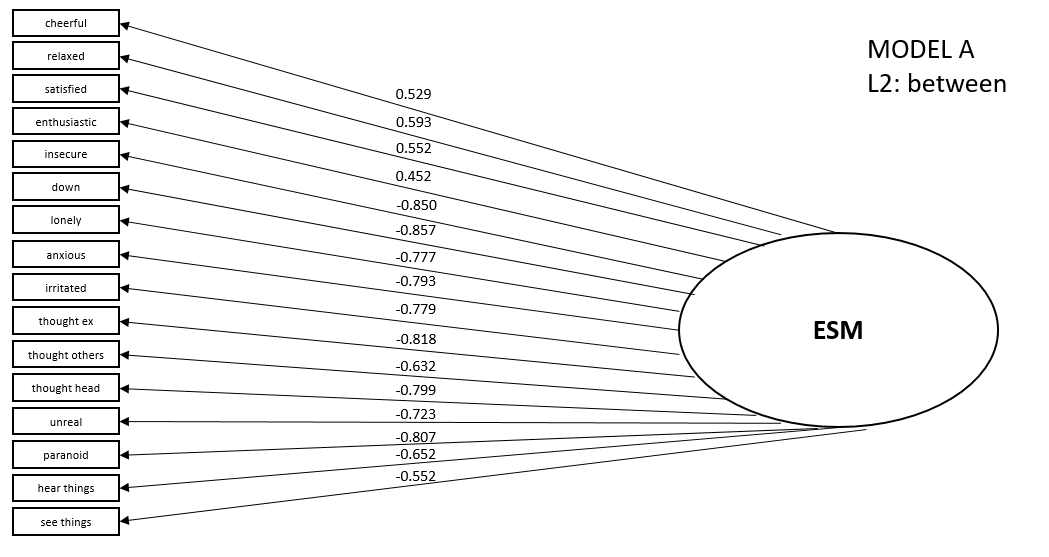


Figure S2. Model A, L2: between.


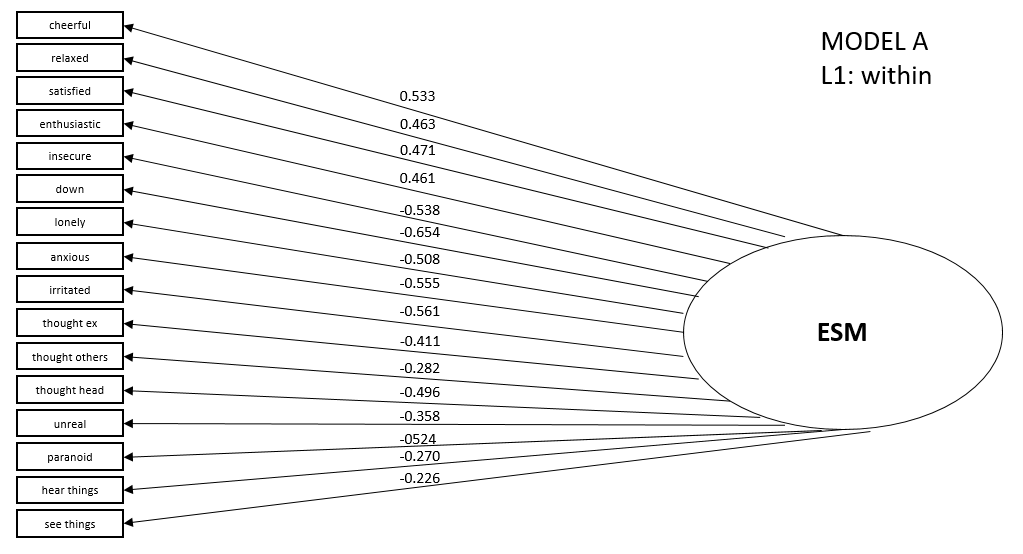
Figure S3. Model A, L 1: within.
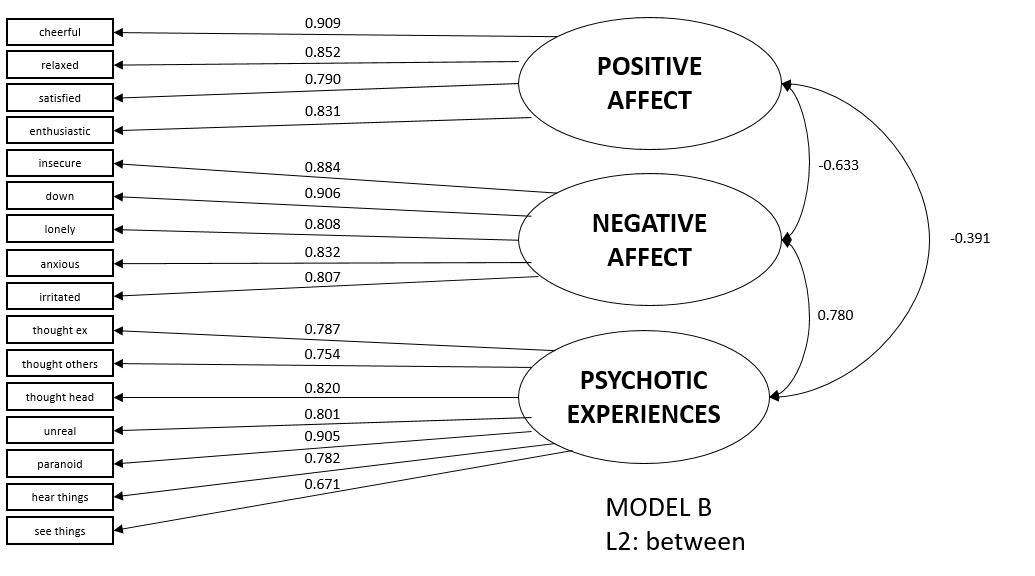


Figure S4. Model B, Level 2: between.


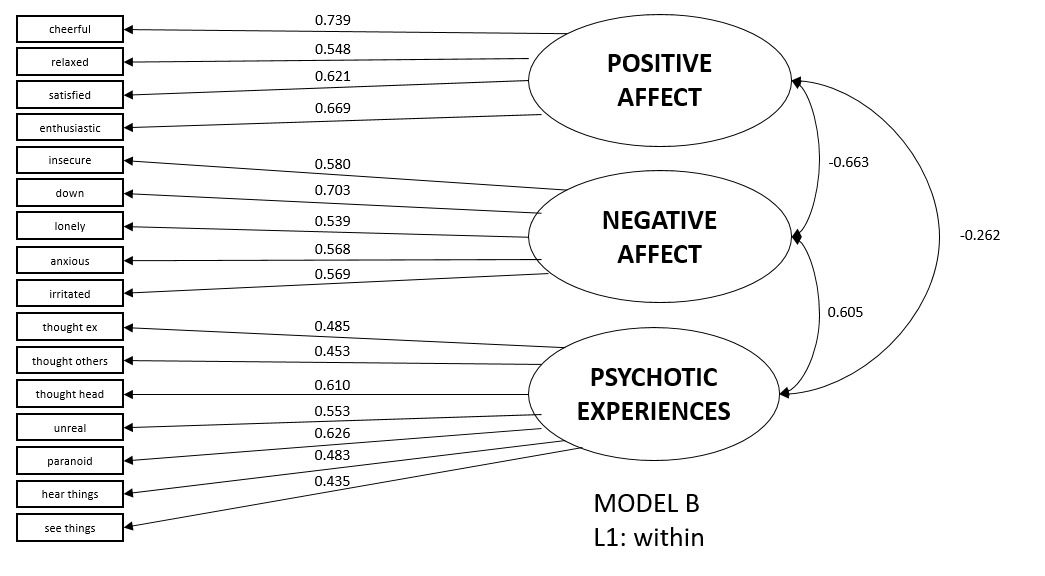


Figure S5. Model B, Level 1: within.

**American Psychiatric Association**. (2002) Multiaxial assessment. *DSM-IV-TR: Diagnostic and Statistical Manual of Mental Disorders.* Washington, DC: American Psychiatric Assoication.

**Bernstein DP, Ahluvalia T, Pogge D and Handelsman L**. (1997) Validity of the Childhood Trauma Questionnaire in an adolescent psychiatric population. *Journal of the American Academy of Child & Adolescent Psychiatry* **36**: 340-348.

**Bernstein DP and Fink L**. (1998) *Childhood trauma questionnaire: A retrospective self-report: Manual,* San Antonio, Texas: The Psychological Corporation.

**Bernstein DP, Stein JA, Newcomb MD, Walker E, Pogge D, Ahluvalia T, Stokes J, Handelsman L, Medrano M and Desmond D**. (2003) Development and validation of a brief screening version of the Childhood Trauma Questionnaire. *Child abuse & neglect* **27**: 169-190.

**Brown TA**. (2006) *Confirmatory factor analysis for applied research*: The Guilford Press.

**Brown TA and Moore MTJHosem**. (2012) Confirmatory factor analysis. 361-379.

**Delespaul P, deVries M and van Os J**. (2002) Determinants of occurrence and recovery from hallucinations in daily life. *Social Psychiatry and Psychiatric Epidemiology* **37**: 97-104.

**Dziak JJ, Coffman DL, Lanza ST, Li R and Jermiin LSJBib**. (2020) Sensitivity and specificity of information criteria. **21**: 553-565.

**Guy W**. (1976) ECDEU assessment manual for psychopharmacology. *US Department of Health, and Welfare*: 534-537.

**Klippel A, Schick A, Myin-Germeys I, Vaessen T and Reininghaus U**. (submitted) Modeling the temporal interplay between stress and affective disturbances in pathways to psychosis: an experience sampling study.

**Myin-Germeys I, Delespaul PH and Van Os J**. (2005) Behavioural sensitization to daily life stress in psychosis. *Psychological Medicine* **35**: 733-741.

**Myin-Germeys I, Peeters F, Havermans R, Nicolson N, DeVries MW, Delespaul P and Van Os J**. (2003) Emotional reactivity to daily life stress in psychosis and affective disorder: an experience sampling study. *Acta Psychiatrica Scandinavica* **107**: 124-131.

**Myin-Germeys I, van Os J, Schwartz JE, Stone AA and Delespaul PA**. (2001) Emotional Reactivity to Daily Life Stress in Psychosis. *Archives of General Psychiatry* **58**: 1137-1144.

**Palmier-Claus J, Dunn G and Lewis S**. (2012) Emotional and symptomatic reactivity to stress in individuals at ultra-high risk of developing psychosis. *Psychological Medicine* **42**: 1003-1012.

**Pries LK, Klingenberg B, Menne-Lothmann C, Decoster J, van Winkel R, Collip D, Delespaul P, De Hert M, Derom C, Thiery E, Jacobs N, Wichers M, Cinar O, Lin BD, Luykx JJ, Rutten BPF, van Os J and Guloksuz S**. (2020) Polygenic liability for schizophrenia and childhood adversity influences daily-life emotion dysregulation and psychosis proneness. *Acta Psychiatrica Scandinavica*.

**R Core Team**. (2019) R: A language and environment for statistical computing. Vienna, Austria: R Foundation for Statistical Computing.

**Reininghaus UA, Gayer-Anderson C, Valmaggia L, Kempton M, Calem M, Onyejiaka A, Hubbard K, Dazzan P, Beards S, Fisher H, Mills J, McGuire P, Craig T, Garety P, van Os J, Murray R, Wykes T, Myin-Germeys I and Morgan C**. (2016) Psychological processes underlying the association between childhood trauma and psychosis in daily life: an experience sampling study. *Psychological Medicine* **46**: 2799-2813.

**Scher CD, Stein MB, Asmundson GJ, McCreary DR and Forde DR**. (2001) The childhood trauma questionnaire in a community sample: psychometric properties and normative data. *Journal of traumatic stress* **14**: 843-857.

**StataCorp**. (2017) Stata Statistical Software: Release 15. College Station, TX: StataCorp LLC.

**Wingenfeld K, Spitzer C, Mensebach C, Grabe HJ, Hill A, Gast U, Schlosser N, Hopp H, Beblo T and Driessen M**. (2010) The German Version of the Childhood Trauma Questionnaire (CTQ): Preliminary Psychometric Properties. *Psychotherapie Psychosomatik Medizinische Psychologie* **60**: e13.

**Yung AR, Yuen HP, McGorry PD, Phillips LJ, Kelly D, Dell'Olio M, Francey SM, Cosgrave EM, Killackey E and Stanford C**. (2005) Mapping the onset of psychosis: The comprehensive assessment of at‐risk mental states. *Australian and New Zealand Journal of Psychiatry* **39**: 964-971.
